# External validation and refinement of the Psychosis Polyrisk Score to detect individuals at clinical high risk for psychosis

**DOI:** 10.1136/bmjment-2026-302838

**Published:** 2026-09-28

**Authors:** Riccardo Stefanelli, Dominic Oliver, Stefano Damiani, Marika Orlandi, Umberto Provenzani, Elisa Bortolin, Margherita Rovida, Jean Addington, Luis Alameda, Celso Arango, Nicholas J Breitborde, Matthew Broome, Kristin S Cadenhead, Monica E Calkins, Ricardo E Carrion, Rolando I Castillo-Passi, Yu Hai Eric Chen, Jimmy Choi, Philippe Conus, Barbara A Cornblatt, Covadonga M Díaz-Caneja, Lauren M Ellman, Pablo A Gaspar, Carla Gerber, Louise Birkedal Glenthøj, Leslie E Horton, Christy LM Hui, Joseph Kambeitz, Lana Kambeitz-Ilankovic, Matcheri S Keshavan, Minah Kim, Sung-Wan Kim, Nikolaos Koutsouleris, Jun Soo Kwon, Kerstin Langbein, Daniel Mamah, Daniel H Mathalon, Vijay A Mittal, Meredete Nordentoft, Godfrey D Pearlson, Nora Penzel, Jesus Perez, Diana O Perkins, Albert R Powers, Jack R Rogers, Fred W Sabb, Jason Schiffman, Jai Shah, Steven M Silverstein, Stefan Smesny, William S Stone, Gregory P Strauss, Judy L Thompson, Rachel Upthegrove, Swapna Verma, Jijun Wang, Daniel H Wolf, TianHong Zhang, Sylvain Bouix, Cheryl M Corcoran, Tina Kapur, Ofer Pasternak, Carrie Bearden, Rene S Kahn, John M Kane, Patrick D McGorry, Barnaby Nelson, Martha E Shenton, Scott W Woods, Accelerating Medicines Partnership Schizophrenia (AMP SCZ), Paolo Fusar-Poli

**Affiliations:** 1 Department of Brain and Behavioral Sciences, University of Pavia, Pavia, Italy; 2 Department of Psychiatry, University of Oxford, Oxford, UK; 3 NIHR Oxford Health Biomedical Research Centre, Oxford, UK; 4 OPEN Early Detection Service, Oxford Health NHS Foundation Trust, Oxford, UK; 5 Department of Psychosis Studies, Early Psychosis: Interventions and Clinical-detection (EPIC) Lab, Institute of Psychiatry, Psychology & Neuroscience, King’s College London, London, UK; 6 Child Neurology and Psychiatry Unit, IRCCS Mondino Foundation, Pavia, Italy; 7 Università Vita-Salute San Raffaele, Milan, Italy; 8 Department of Psychiatry, Hotchkiss Brain Institute, University of Calgary, Alberta, Canada; 9 TiPP Program Department of Psychiatry, Service of General Psychiatry, Lausanne University Hospital, Lausanne, Switzerland; 10 Department of Psychiatry, Centro Investigación Biomedica en Red de Salud Mental (CIBERSAM), Instituto de Biomedicina de Sevilla (IBIS), Hospital Universitario Virgen del Rocío, University of Sevilla, Sevilla, Spain; 11 Department of Psychosis Studies, Institute of Psychiatry, Psychology and Neuroscience, King's College London, London, UK; 12 Department of Child and Adolescent Psychiatry, Hospital Universitario La Paz, IdiPAZ, CIBERSAM, School of Medicine, Universidad Autónoma de Madrid, Madrid, Spain; 13 Department of Psychiatry and Behavioral Health, The Ohio State University, Columbus, Columbus, OH, USA; 14 School of Psychology, Institute for Mental Health, University of Birmingham, Birmingham, UK; 15 Early Intervention for Psychosis Services, Birmingham Women's and Children's NHS Foundation Trust, Birmingham, UK; 16 Department of Psychiatry, University of California, San Diego, CA, USA; 17 Department of Psychiatry, Perelman School of Medicine, University of Pennsylvania, Philadelphia, PA, USA; 18 Northwell Health, New Hyde Park, New York, USA; 19 Department of Psychiatry, Donald and Barbara Zucker School of Medicine at Hofstra/Northwell, Hempstead, NY, USA; 20 Institute of Behavioral Science, Feinstein Institutes for Medical Research, Northwell Health System, Manhasset, NY, USA; 21 Department of Psychiatry, University of Chile, University of Chile, Santiago, Chile; 22 Department of Neurology, Psychology and Psychiatry, Clínica Alemana-Universidad del Desarrollo, Santiago, Chile; 23 Department of Psychiatry, University of Hong Kong, Pok Fu Lam, Hong Kong; 24 Olin Neuropsychiatry Research Center, Hartford HealthCare, Hartford, CT, USA; 25 Department of Child and Adolescent Psychiatry, Institute of Psychiatry and Menthal Health, Hospital General Universitario Gregorio Maranon, IiSMG, CIBERSAM, Instituo de Salud Carlos III, School of Medicine, Universidad Complutense, Madrid, Spain; 26 Fundación para la Investigación Biomédica del Hospital Universitario La Paz, Madrid, Spain; 27 Department of Psychology & Neuroscience, Temple University, Philadelphia, PA, USA; 28 Department of Psychiatry, University of Chile, Santiago, Chile; 29 Behavioral Health Services, PeaceHealth Medical Group, Eugene, OR, USA; 30 VIRTU Research Group, Mental Health Copenhagen, University of Copenhagen, Copenhagen, Denmark; 31 Department of Psychology, University of Copenhagen, Copenhagen, Denmark; 32 Department of Psychiatry, University of Pittsburgh School of Medicine, Pittsburgh, PA, USA; 33 Department of Psychiatry, Faculty of Medicine and University Hospital Cologne, University of Cologne, Cologne, Germany; 34 Department of Psychiatry, Beth Israel Deaconess Medical Center and Harvard Medical School, Boston, MA, USA; 35 Department of Neuropsychiatry, Seoul National University Hospital, Seoul, Republic of Korea; 36 Department of Psychiatry, Seoul National University College of Medicine, Seoul, Republic of Korea; 37 Institute of Human Behavioral Medicine, SNU-MRC, Seoul, Republic of Korea; 38 Department of Psychiatry, Chonnam National University Medical School, Gwangju, Korea; 39 Mindlink, Gwangju Bukgu Mental Health Center, Gwangju, Korea; 40 Department of Psychiatry and Psychotherapy, Ludwig Maximilian University of Munich, Munich, Germany; 41 Department of Psychiatry, Hanyang University College of Medicine, Seoul, Korea; 42 Department of Neuropsychiatry, Hanyang University Hospital, Seoul, Korea; 43 Institute of Human Behavioral Medicine, SNU-MRCInstitute of Human Behavioral Medicine, SNU-MRC, Seoul, Republic of Korea; 44 Department of Psychiatry and Psychotherapy, Jena University Hospital, Jena, Germany; 45 Department of Psychiatry, Washington University Medical School, St. Louis, MO, USA; 46 Department of Psychiatry and Behavioral Sciences and Weill Institute for Neurosciences, University of California, San Francisco, CA, USA; 47 Mental Health Service 116D, Veterans Affairs San Francisco Health Care System, San Francisco, CA, USA; 48 Department of Psychology, Northwestern University, Evanston, IL, USA; 49 Copenhagen Research Centre for Mental Health, Mental Health Copenhagen, University of Copenhagen, Copenhagen, Denmark; 50 Department of Clinical Medicine, University of Copenhagen, Copenhagen, Denmark; 51 Department of Psychiatry, Yale University School of Medicine, New Haven, CT, USA; 52 Department of Psychiatry, Massachusetts General Hospital and Harvard Medical School, Boston, MA, USA; 53 CAMEO, Early Intervention in Psychosis Service, Cambridgeshire and Peterborough NHS Foundation Trust, Cambridge, UK; 54 Institute of Biomedical Research (IBSAL), Department of Medicine, Universidad de Salamanca, Salamanca, Spain; 55 Department of Psychiatry, University of North Carolina at Chapel Hill, Chapel Hill, NC, USA; 56 Connecticut Mental Health Center, New Haven, Connecticut, USA; 57 Centre for Human Brain Health, School of Psychology, University of Birmingham, Birmingham, UK; 58 Prevention Science Institute, University of Oregon, Eugene, OR, USA; 59 Department of Psychological Science, University of California, Irvine, CA, USA; 60 Department of Psychiatry, McGill University, Montreal, Quebec, Canada; 61 PEPP-Montreal, Douglas Research Centre, Montreal, Quebec, Canada; 62 Department of Psychiatry, University of Rochester Medical Center, Rochester, NY, USA; 63 Department of Ophthalmology, University of Rochester Medical Center, Rochester, NY, USA; 64 Department of Neuroscience, University of Rochester Medical Center, Rochester, NY, USA; 65 Department of Psychology, University of Georgia, Athens, GA, USA; 66 Departments of Psychiatry and Neuroscience, University of Rochester Medical Center, Rochester, NY, USA; 67 Department of Psychosis, Institute of Mental Health, Singapore; 68 Shanghai Mental Health Center, Shanghai Jiaotong University School of Medicine, Shanghai, China; 69 Shanghai Mental Health Center, Shanghai Jiaotong University School of Medicine, Neuromodulation Center, Shanghai Engineering Research Center of Intelligent Psychological Evaluation and Intervention, Shanghai Key Laboratory of Psychotic Disorders, Shangai, China; 70 Department of Software Engineering and Information Technology, École de technologie supérieure, Montréal, Quebec, Canada; 71 Department of Psychiatry, Icahn School of Medicine at Mount Sinai, New York, NY, USA; 72 Department of Radiology, Brigham and Women's Hospital and Harvard Medical School, Boston, MA, USA; 73 Department of Psychiatry, Mass General Brigham Hospital, Harvard Medical School, Boston, MA, USA; 74 Department of Radiology, Mass General Brigham Hospital, Harvard Medical School, Boston, MA, USA; 75 Department of Psychiatry and Biobehavioral Sciences, Semel Institute for Neuroscience and Human Behavior, University of California, Los Angeles, Los Angeles, CA, USA; 76 Department of Psychology, Semel Institute for Neuroscience and Human Behavior, University of California, Los Angeles, Los Angeles, CA, USA; 77 Institute of Behavioral Science, Feinstein Institutes for Medical Research, Northwell Health, Manhasset, NY, USA; 78 Centre for Youth Mental Health, The University of Melbourne, Melbourne, Victoria, Australia; 79 Orygen, The National Centre of Excellence in Youth Mental Health, Melbourne, Victoria, Australia; 80 Department of Psychiatry, Brigham and Women's Hospital and Harvard Medical School, Boston, MA, USA; 81 Outreach and Support in South-London (OASIS) service, South London and Maudsley (SLaM) NHS Foundation Trust, London, UK

**Keywords:** Psychiatry, Schizophrenia Spectrum and Other Psychotic Disorders, Behavioral Medicine

## Abstract

**Background:**

The Psychosis Polyrisk Score (PPS) was developed to characterise exposure to environmental and developmental risk factors for psychosis. In previous studies, the PPS showed promise particularly in discriminating between individuals at clinical high risk for psychosis (CHR-P) and community controls (CC).

**Objectives:**

This study aimed to (1) perform an external validation of the PPS model in a large independent sample and (2) refine the model using expanded predictor data.

**Methods:**

Participants were recruited through the AMP SCZ program: n=1642 CHR-P, n=519 CC. We performed a formal external validation of the PPS original model. The model was then refined incorporating expanded predictor data (perceived loneliness, neighbourhood quality) and increasing the granularity of existing predictors (ethnicity, childhood trauma, parental severe mental illness). Next, we evaluated the refined model through repeated nested cross-validation and compared it to the original model retrained in the new dataset. Discrimination (Harrell’s C-index) and calibration (intercept and slope) were used to assess model performance.

**Findings:**

The externally validated PPS original model showed good discrimination (C=0.777, 95% CI 0.756 to 0.797) between CHR-P and CC and good calibration (intercept=0; slope=1.003) after recalibration. The refined PPS model exhibited higher internally cross-validated discrimination than the retrained original model (C=0.876, 95% CI 0.860 to 0.891 vs C=0.796, 95% CI 0.775 to 0.816), as well as good calibration performance (intercept=−0.008; slope=1.031).

**Conclusions:**

The PPS showed good generalisability to a large, independent sample, and performance improved after model refinement.

**Clinical implications:**

The PPS represents a potential scalable tool within stepped assessment frameworks for identifying individuals who warrant further CHR-P assessment.

**Trial registration number:**

NCT05905003.

**WHAT IS ALREADY KNOWN ON THIS TOPIC**

- The Psychosis Polyrisk Score (PPS) integrates environmental and developmental risk factors for psychosis and has shown promise for discriminating individuals at clinical high risk for psychosis from controls, but its performance in a large independent cohort requires evaluation.

**WHAT THIS STUDY ADDS**

- The PPS original model generalises well to the large Accelerating Medicines Partnership Schizophrenia (AMP SCZ) validation cohort. Refining the model with expanded and more granular predictor data results in additional performance improvement.

**HOW THIS STUDY MIGHT AFFECT RESEARCH, PRACTICE OR POLICY**

- These findings support further evaluation of the PPS within stepped CHR-P assessment pathways, particularly in enriched or help-seeking settings, while highlighting the need for external validation of the refined model and longitudinal testing for transition to psychosis.

## Introduction

 Early detection of individuals at clinical high risk for psychosis (CHR-P) is central to preventive psychiatry, given its potential to improve outcomes and reduce the societal burden of psychotic disorders.^1^ However, detection of CHR-P individuals is suboptimal. In clinical services, individuals with a first episode of psychosis (FEP) detected during their CHR-P stage are currently 5–14%,^2–4^ despite 80% experiencing a detectable prodromal phase.^5 6^ This issue is mirrored following psychosis onset, with more than half of FEP patients being undetected by current early intervention frameworks.^7^ Given that recruitment strategies have a substantial impact on the level of risk within CHR-P samples,^8–10^ it is crucial to develop tools that can facilitate large-scale screening without diluting risk.

Psychosis risk can be conceptualised as the cumulative effect of multiple environmental and developmental factors that alter underlying neurobiology, whose aggregation may be more informative than any single exposure or symptom alone.^10–12^ Within this framework, the Psychosis Polyrisk Score (PPS)^10^ was developed as a translational self-report instrument that integrates multiple environmental risk and protective factors based on the umbrella review by Radua *et al*^12^ into a single composite score, mirroring the logic of the polygenic risk score (PRS).^10 11 13^ The PPS captures key domains of environmental risk, including sociodemographic factors, migration status, childhood adversity, substance use, parental psychopathology and residential context (eg, urbanicity, pollution and ethnic density). As a scalable self-report tool, the PPS is intended to complement symptom-based prescreening instruments such as the 16-item Prodromal Questionnaire (PQ-16).^14^

In the European Network of Schizophrenia Networks for the Study of Gene-Environment Interactions (EU-GEI) study, the PPS explained over 30% of the variance between CHR-P individuals and healthy controls, compared with <2% for the PRS.^15 16^ Moreover, in formal prediction modelling analyses, the PPS showed excellent discrimination (Harrell’s C ≈ 0.91) between CHR-P and healthy controls in internal validation and displayed potential clinical net benefit of use in clinical samples.^16^ These results highlight the potential of the PPS as a scalable, pragmatic tool for CHR-P identification. However, there is a need for replication in independent, larger and more diverse populations to establish robustness and generalisability.

Thus, the present study used the Accelerating Medicines Partnership Schizophrenia (AMP SCZ) program dataset^17^ to (1) externally validate the PPS original model developed from the EU-GEI study and (2) refine the model using expanded predictor data based on a recent update of Radua and colleagues’ umbrella review.^18^ The focus of both aims was discrimination between CHR-P individuals and a sample of community controls (CC).

## Methods

### Sample

Participants were drawn from the AMP SCZ programme, which included the Psychosis-Risk Outcomes Network and the Prediction Scientific Global Consortium Research Networks, for a total of 38 sites across the globe.^17^ The project was approved by the governing institutional review board at each site. Participants and/or their legal representatives provided informed consent and/or assent as appropriate. For a broader overview of AMP SCZ procedure and structure, see Wannan *et al*.^17^

Analyses were conducted using AMP SCZ data Release V.3.0, which included de-identified baseline data from n=1642 individuals meeting CHR-P criteria and n=519 CC.^19^ For sample size calculation and inclusion/exclusion criteria, see online supplemental eMethods.

### Measures

The primary outcome was group status (CHR-P vs CC) at baseline (ie, a cross-sectional classification outcome, with no prospective time horizon). We evaluated two sets of predictors.

1. *Predictor set 1* (PPS original model): predictors of the PPS original model developed by Oliver *et al* using the EU-GEI dataset.^16^ These predictors included: age, gender, ethnicity (white, black, other), handedness, first-generation and second-generation migrant status, North African immigrant background, paternal socioeconomic status, parental history of severe mental illness, recent adult stressful life events, daily tobacco use, weekly cannabis use, childhood trauma (total score only) and trait anhedonia.
2. *Predictor set 2* (PPS refined model): an expanded predictor set aiming to improve the PPS original model, based on the update of Radua and colleagues’ review on psychosis risk/protective factors.^12 18^ Here, some variables were defined with higher granularity, including ethnicity (South Asian, East Asian, Southeast Asian, West-Central Asia/Middle-Eastern, Indigenous-Aboriginal, Mixed, Pacific-Islander), childhood trauma (sexual abuse, physical abuse, emotional abuse, physical neglect, emotional neglect) and parental history of severe mental illness (mother/father psychosis, mother/father other severe mental illness). Similarly, weekly cannabis use was replaced by daily cannabis use (to capture heavier use), and two new predictors were added: perceived loneliness and neighbourhood quality. Lastly, childhood trauma and anhedonia were considered as continuous predictors instead of using cutoffs as in predictor set 1.

The full list of predictors, along with the instruments used to derive each of them, can be found in the online supplemental material (online supplemental etable 1).

### Procedure

The analytical approach proceeded in two steps:

1. *External validation*: for this step, the logistic regression coefficients estimated during model development using EU-GEI data were applied to predictor data (predictor set 1) from AMP SCZ. The aim was to assess the extent to which this original, already-developed model performed when applied to new, unseen data.
2. *Model refinement*: this step aimed to evaluate whether the inclusion of additional predictors (predictor set 2) enhanced model discrimination and calibration relative to the original model. However, due to the expected performance drop when externally validating a model,^20^ direct comparison with the external validation result above would provide an implicit advantage to the refined model. We therefore retrained the PPS original model within the AMP SCZ dataset (predictor set 1), estimating new coefficients.

To improve conceptual clarity, we added a schematic overview of the analytical pipeline illustrating the distinction between the external validation of the PPS original model, the retrained original model and the refined model (figure 1).

**Figure 1:**
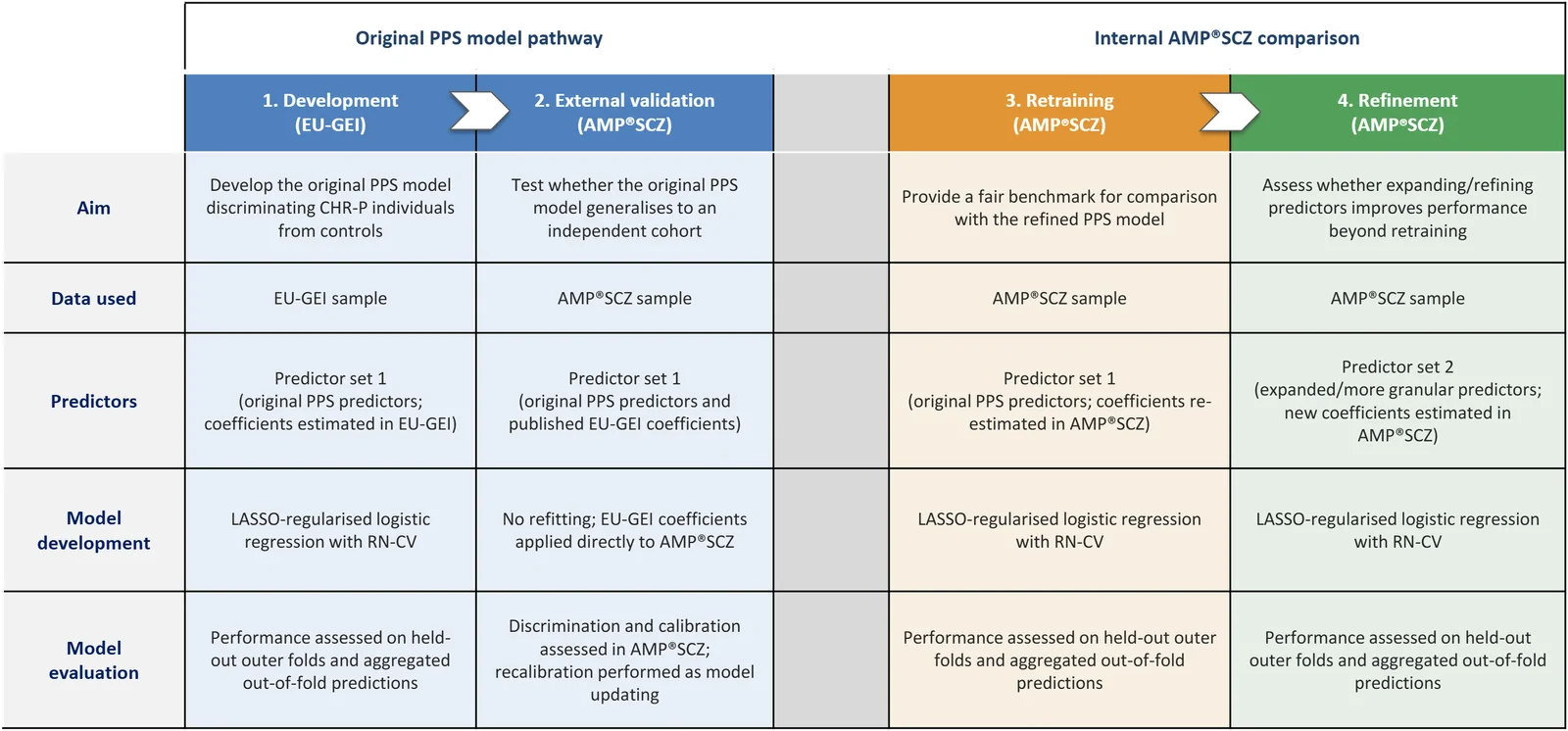
Analytical pipeline of PPS development, external validation, retraining and refinement*. The figure summarises the four analytic stages linking the original EU-GEI PPS model and the present AMP SCZ analyses. First, the original PPS model was developed in the EU-GEI sample using predictor set 1 and Least Absolute Shrinkage and Selection Operator (LASSO)-regularised logistic regression with repeated nested cross-validation. Second, the published EU-GEI coefficients were applied directly to the independent AMP SCZ sample for external validation of the original PPS model. Third, to provide a fair benchmark for model refinement, the original predictor set was retrained within AMP SCZ using the same repeated nested cross-validation framework. Fourth, the refined PPS model was developed within AMP SCZ using an expanded and more granular predictor set. The external validation analysis is presented separately from the internal AMP SCZ comparison, where the main comparison is between the retrained original model and the refined model. *Key distinction: the refined model is compared against the retrained original model within AMP SCZ; the external validation procedure is shown separately because it reflects independent evaluation of the original PPS in a new validation cohort. AMP SCZ, Accelerating Medicines Partnership Schizophrenia; CHR-P, clinical high risk for psychosis; EU-GEI, European Network of National Schizophrenia Networks Studying Gene-Environment Interactions; PPS, Psychosis Polyrisk Score; RN-CV, repeated nested cross-validation.

### Statistical analyses

Reporting was guided by the Transparent Reporting of a multivariable prediction model for Individual Prognosis Or Diagnosis statement,^21^ and analyses were conducted in line with contemporary methodological guidance for prediction-model evaluation and updating.^20^ All analyses were performed in R (V.4.2). Analytic codes for model development and validation are available online (https://github.com/AMPSCZ).

1. *External validation of the PPS original model (predictor set 1*): the logistic regression coefficients from the EU-GEI-based PPS model^16^ were directly applied to the AMP SCZ dataset to compute individual-level predicted probabilities of CHR-P status. These were obtained by multiplying each coefficient calculated in the EU-GEI study by the raw score of the variables within the model. Variables with missingness >20% were excluded, whereas missing data <20% were imputed using random forest imputation (missForest R package, V.1.6.1). Primary outcomes were discrimination and calibration. Discrimination refers to the model’s ability to correctly distinguish between individuals with and without the outcome (ie, CHR-P vs CC), whereas calibration assesses how closely the predicted probabilities align with the observed outcomes. Discrimination was evaluated using Harrell’s C with 95% CIs obtained via 2000 bootstrap resamples. Calibration was assessed through the calibration intercept and slope, the Brier score and the Integrated Calibration Index (ICI). Threshold-dependent classification performance (ie, balanced accuracy, sensitivity, specificity, positive predictive value and negative predictive value) was measured across 100 evenly spaced probability cut-offs (0.01–1.00). The threshold yielding the highest balanced accuracy was chosen to report final performance metrics. To account for case-mix differences between the EU-GEI and AMP SCZ samples, the model was recalibrated by refitting a logistic regression with the PPS score as the sole covariate, thereby updating the intercept and slope to the new data distribution. Model performance was re-evaluated post-recalibration in the same way as above.
2. *PPS model refinement and internal validation (predictor set 2*): we fitted a refined version of the model using Least Absolute Shrinkage and Selection Operator (LASSO)-regularised logistic regression (glmnet R package, V.4.1.10) and repeated nested cross-validation (RN-CV). This technique framework combines two layers of cross-validation: an inner loop for hyperparameter tuning and model selection, and an outer loop for evaluating model performance on unseen data. By repeating this process multiple times with different random splits, RN-CV provides robust estimates of generalisation performance and minimises overfitting and optimism bias.^22^ In the present study, each RN-CV procedure employed five outer folds and five inner folds, repeated 10 times to ensure stability, mirroring the procedure of Oliver *et al*.^16^ Within each outer training fold, predictors with >20% missing values were excluded. The remaining missing data were imputed through random forest separately from any outcome data and independently in the training and test sets, ensuring no data leakage across folds. All predictors were standardised. Model performance was evaluated on the held-out outer test folds, yielding 50 out-of-fold estimates per metric (five outer folds×10 repeats). Out-of-fold predictions were then aggregated at the subject level by averaging predicted probabilities across repetitions. Overall model performance was computed on these aggregated predictions, and 95% CIs were estimated from empirical bootstrap distributions obtained through subject-level resampling (2000 iterations). Model performance was further evaluated across sites to ensure stability (leave-one-site-out cross validation). Sensitivity analyses were conducted to assess whether performance of the refined PPS model was dependent on predictors potentially affected by eligibility criteria, recruitment procedures or concerns regarding fairness and generalisability. First, we refitted the refined model after excluding age and parental psychosis, as CC were intended to be matched to CHR-P participants on age and were excluded if they had a full-blown first-degree relative with a psychotic spectrum disorder (as such, these predictors could potentially artificially inflate discrimination performance). Second, we refitted the model after excluding gender and ethnicity, given that these are sensitive sociodemographic predictors and, particularly for ethnicity, may partly reflect site composition or recruitment patterns in a large international multisite dataset. These predictors were retained in the main model because of their relevance in the psychosis-risk literature^12 18^ as well as potential importance for future external validations.

Next, to enable appropriate comparison between the refined and the original PPS model, we retrained the original model using predictor set one in the new AMP SCZ dataset without a priori coefficients, using the same RN-CV procedure outlined above.

We finally computed the logistic regression coefficients of the refined PPS model by fitting a final fivefold cross-validation on the full AMP SCZ raw dataset. These coefficients were used to compute new individual PPS scores, which were then correlated with subthreshold positive symptom severity (assessed using the PSYCHS) and socio-occupational functioning (assessed using the Social and Occupational Functioning Assessment Scale (SOFAS). Spearman’s *ρ* coefficient was used to account for non-normal distributions. Predictors with missingness >20% and coefficients shrunk to zero were excluded from the analysis. Standardised model coefficients with continuous variables were multiplied by two SD to increase comparability of variable importance across categorical and continuous predictors.

The same procedure was used to compute the logistic regression coefficients of the retrained PPS original model.

Lastly, we used decision curve analysis to evaluate the clinical utility of each model by estimating the net benefit of PPS-based classification across a range of threshold probabilities. These represent the minimum predicted probability of meeting CHR-P criteria at which a clinician would decide to refer an individual for further CHR-P assessment. For instance, a threshold of 0.20 implies that the potential benefit of assessing someone with a 20% risk outweighs the cost of assessing someone who ultimately does not meet CHR-P criteria. In this context, ‘treatment’ refers to offering a CHR-P clinical assessment (eg, Comprehensive Assessment of At-Risk Mental States (CAARMS)/Structured Interview for Psychosis-Risk Syndromes (SIPS)), rather than initiating any therapeutic intervention. We assessed clinical utility in clinical populations and the general population using threshold probabilities (clinical: 0–0.5; general: 0–0.3) and the meta-analytic prevalence of CHR-P in the relevant population (general: 1.72%; clinical: 19.2%).^23^ These two scenarios were used as lower-bound and upper-bound estimates, respectively, because the intended stepped-assessment use case is likely enriched relative to the general population but less enriched than specialist CHR-P clinical samples. The analysis was implemented using the dcurves package (V.0.5.1).

## Results

### Sample

Most participants were assigned the female sex at birth, both within the CHR-P group (64.4%) and the CC group (61.5%). The mean age was 21.2±4.0 years for CHR-P individuals and 21.6±3.6 for CC. Most of CC (60.9%) and CHR-P individuals (54.8%) identified as females (gender), but within the CHR-P group a proportion of individuals were also non-binary (8.2%) or identified with other genders (1.8%). As for ethnicity, the majority was white (CC=52.3%, CHR-P=58.3%), with a fairly equal representation of the other ethnicities across the sample.

All predictor statistics can be found in table 1, whereas for a more in-depth overview of participants’ demographics and comorbidities see Addington *et al*.^19^

**Table 1:** Mean, SD, percentage and missing data of all candidate predictors considered for the refined PPS model (predictor set 2)

	CC	CHR-P	Missing CC	Missing CHR-P
	n=519	n=1642	N (%)	N (%)
Age, years	21.6 (3.6)	21.2 (4.0)	19 (3.7%)	62 (3.8%)
Paternal age, years	32.4 (10.8)	34.5 (8.4)	192 (37.0%)	816 (49.7%)
Gender				
Female	313 (60.9%)	884 (54.8%)	5 (1.0%)	29 (1.8%)
Male	193 (37.5%)	568 (35.2%)	5 (1.0%)	29 (1.8%)
Non-binary	8 (1.6%)	132 (8.2%)	5 (1.0%)	29 (1.8%)
Other	0 (0%)	29 (1.8%)		29 (1.8%)
Ethnicity				
Black	32 (6.2%)	137 (8.6%)	7 (1.3%)	56 (3.4%)
East Asian	91 (17.8%)	138 (8.7%)	7 (1.3%)	56 (3.4%)
Indigenous Aboriginal	3 (0.6%)	19 (1.2%)	7 (1.3%)	56 (3.4%)
Mixed	34 (6.6%)	141 (8.9%)	7 (1.3%)	56 (3.4%)
Pacific Islander	0 (0%)	5 (0.3%)		56 (3.4%)
South Asian	49 (9.6%)	78 (4.9%)	7 (1.3%)	56 (3.4%)
South East Asia	27 (5.3%)	92 (5.8%)	7 (1.3%)	56 (3.4%)
West Central Asia or Middle Eastern	8 (1.6%)	52 (3.3%)	7 (1.3%)	56 (3.4%)
White	268 (52.3%)	924 (58.3%)	7 (1.3%)	56 (3.4%)
Childhood trauma	35.2 (10.7)	52.0 (16.6)	66 (12.7%)	257 (15.7%)
Emotional abuse	8.3 (4.0)	14.2 (5.8)	66 (12.7%)	257 (15.7%)
Physical abuse	5.8 (2.1)	7.7 (3.9)	66 (12.7%)	261 (15.9%)
Sexual abuse	5.7 (2.3)	7.6 (4.3)	66 (12.7%)	261 (15.9%)
Emotional neglect	9.1 (4.2)	13.7 (5.0)	66 (12.7%)	257 (15.7%)
Physical neglect	6.4 (2.1)	8.8 (3.4)	66 (12.7%)	257 (15.7%)
Loneliness	8.2 (2.2)	12.2 (3.0)	66 (12.7%)	260 (15.8%)
Neighbourhood quality	36.9 (5.7)	33.8 (6.3)	66 (12.7%)	255 (15.5%)
Trait anhedonia	43.5 (11.6)	51.8 (13.7)	66 (12.7%)	257 (15.7%)
Premorbid IQ	110.0 (11.5)	108.1 (14.5)	101 (19.5%)	285 (17.4%)
Childhood social withdrawal	N/A	1.9 (2.1)	519 (100.0%)	561 (34.2%)
Left handedness	7.5%	9.9%	67 (12.9%)	253 (15.4%)
Immigration				
First generation immigrant	22.5%	14.8%	7 (1.3%)	18 (1.1%)
Second generation immigrant	17.0%	16.4%	7 (1.3%)	18 (1.1%)
First generation immigrant from North Africa	0.2%	0.1%	0 (0.0%)	0 (0.0%)
Second generation immigrant from North Africa	0.4%	0.2%	0 (0.0%)	0 (0.0%)
Parental socioeconomic status	5.9%	16.0%	9 (1.7%)	59 (3.6%)
Parental mental illness				
Mother psychosis	0.6%	10.1%	56 (10.8%)	215 (13.1%)
Mother other mental illness	9.9%	33.1%	56 (10.8%)	215 (13.1%)
Father psychosis	0.2%	5.8%	56 (10.8%)	217 (13.2%)
Father other mental illness	4.1%	17.5%	56 (10.8%)	217 (13.2%)
Adult stressful life events	44.5%	74.2%	54 (10.4%)	195 (11.9%)
Daily tobacco	5.7%	13.8%	26 (5.0%)	113 (6.9%)
Daily cannabis	3.9%	11.5%	26 (5.0%)	113 (6.9%)

CC, community controls; CHR-P, clinical high risk for psychosis.

Lastly, online supplemental etable 2 provides an overview comparing the EU-GEI development dataset and the AMP SCZ validation dataset.

### External validation of the PPS original model

The model demonstrated good discrimination in external validation (C-Index=0.777, 95% CI 0.756 to 0.797) (table 2; figure 2). However, calibration was suboptimal (intercept=−0.984; slope=0.947), indicating systematic overestimation of predicted probabilities (online supplemental etable 3 and efigure 1). After recalibration, calibration metrics improved (intercept=0; slope=1.003) (table 2, online supplemental efigure 2). Classification metrics across the 100 thresholds, for both the original and recalibrated model, can be found in the online supplemental material (online supplemental etable 4 and etable 5). No variables needed to be excluded from this analysis due to missingness above 20% (online supplemental etable 6). Online supplemental etable 7 reports subgroup performance of the externally validated PPS model by gender and ethnicity.

**Table 2:** Performance metrics across the three PPS models

	Externally validated PPS original model*		Retrained PPS original model†		Refined PPS model‡	
	Mean	CI	Mean	CI	Mean	CI
C-Index	0.777	(0.756 to 0.797)	0.796	(0.775 to 0.816)	0.876	(0.860 to 0.891)
Balanced accuracy	0.714	(0.694 to 0.734)	0.601	(0.584 to 0.620)	0.748	(0.725 to 0.770)
Sensitivity	0.620	(0.595 to 0.644)	0.974	(0.966 to 0.981)	0.928	(0.915 to 0.940)
Specificity	0.810	(0.755 to 0.844)	0.230	(0.195 to 0.265)	0.569	(0.525 to 0.610)
PPV	0.911	(0.896 to 0.926)	0.800	(0.783 to 0.817)	0.872	(0.856 to 0.887)
NPV	0.402	(0.385 to 0.420)	0.735	(0.665 to 0.801)	0.714	(0.670 to 0.756)
Calibration intercept	0§	(−0.049 to 0.048)	−0.003	(−0.104 to 0.104)	−0.008	(−0.124 to 0.113)
Calibration slope	1.003§	(0.891 to 1.128)	1.193	(1.064 to 1.333)	1.031	(0.939 to 1.122)
Brier score	0.151§	(0.145 to 0.157)	0.143	(0.135 to 0.151)	0.114	(0.106 to 0.123)
ICI	0.299§	(0.292 to 0.307)	0.023	(0.011 to 0.035)	0.018	(0.009 to 0.029)

* Performance metrics of the external validation of the original PPS model (predictor set 1), based on the logistic regression coefficients derived from the EU-GEI study and tested on the AMP SCZ dataset.

† Performance metrics of the retrained PPS original model (predictor set 1), with no a priori coefficients.

‡ Performance metrics of the refined PPS model (predictor set 2).

§ Recalibrated values.

AMP SCZ, Accelerating Medicines Partnership Schizophrenia; EU-GEI, European Network of National Schizophrenia Networks Studying Gene-Environment Interactions; ICI, Integrated Calibration Index; PPS, Psychosis Polyrisk Score.

**Figure 2:**
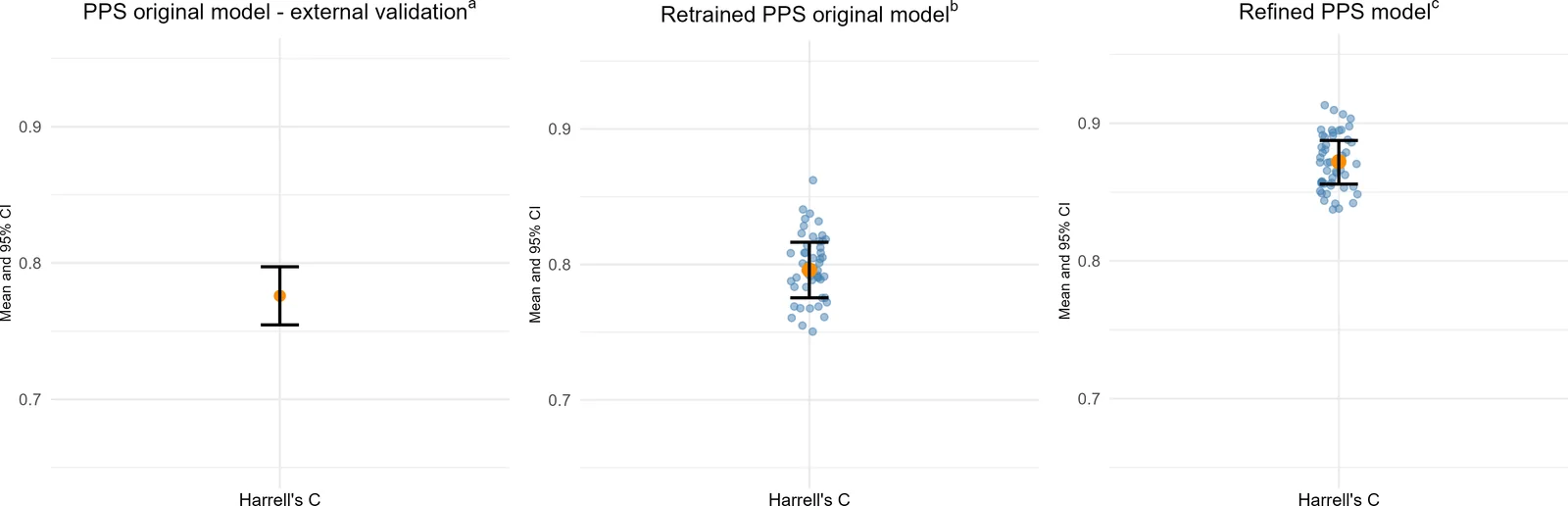
Harrell’s C and 95% CI for each of the three PPS models*. ^a^Harrell’s C mean and CI of the PPS original model (predictor set 1), resulting from the external validation of the logistic regression coefficients derived from the EU-GEI study and tested on the AMP SCZ dataset. ^b^Harrell’s C mean and CI of the retrained PPS original model (predictor set 1), with no a priori coefficients. ^c^Harrell’s C mean and CI of the refined PPS model (predictor set 2). *In both the retrained and refined PPS models, the blue dots are based on the outer-fold estimates from the repeated-nested cross-validation. Each blue dot thus represents the model’s performance on one held-out test fold (5 folds x 10 repeats = 50 estimates, or blue dots). The orange dot and error bars, on the other hand, come from the subject-level bootstrap. For this, the out-of-fold predictions were first aggregated per subject, and then subjects were resampled with replacement to estimate the distribution of the final Harrell’s C and its 95%CI (orange dot). AMP SCZ, Accelerating Medicines Partnership Schizophrenia; EU-GEI, European Network of National Schizophrenia Networks Studying Gene-Environment Interactions; PPS, Psychosis Polyrisk Score.

### Refinement of the PPS original model

The refined model demonstrated excellent performance (C-Index=0.876, 95% CI 0.860 to 0.891), with well-calibrated predictions (intercept=−0.008; slope=1.031) (table 2, figure 2, online supplemental efigure 2). Paternal age and childhood social withdrawal were excluded due to missingness >20% per outer fold (online supplemental etable 8). Discrimination performance was 0.08 higher in the refined model compared with the retrained original model (C-Index=0.796, 95% CI 0.775 to 0.816) with evidence of improved calibration slope (retrained model’s slope=1.193). Full, per-fold classification and calibration indices of the refined and retrained model can be found in the Supplementary Material (online supplemental etables 9–12, respectively). Online supplemental etable 13 reports subgroup performance of the refined PPS model by gender and ethnicity.

Model discrimination varied across sites, with greater variability observed in smaller sites, while larger sites showed more stable performance (mean C-Index=0.884±0.089) (online supplemental etable 14). One site (Melbourne) contributed to a large proportion of participants (n=677). When removing age and parental psychosis, performance remained unchanged (C-Index=0.873, 95% CI 0.856 to 0.889) (online supplemental etable 15).

The predictors associated with the largest increase in being identified as CHR-P were loneliness and paternal psychosis (with betas >1.5). Negative coefficients were observed for South Asian (β=–0.62) and East Asian ethnicity (β=–0.32), though the absolute effect sizes were modest relative to positive predictors. The full list of coefficients and dropped variables are reported in online supplemental etable 16 and etable 17, respectively. The coefficients of the PPS original model used for the external validation, as well as its retrained version, are displayed in online supplemental eTable 18.

Finally, the PPS scores derived from the new logistic regression coefficients showed a weak association with the severity of subthreshold positive symptoms within the CHR-P group (ρ=0.238, p<0.001), and a negative association of similar magnitude with socio-occupational functioning (ρ=-.254, p<0.001) (online supplemental efigure 3).

### Decision curve analysis

In clinical samples, the externally validated PPS model provided no incremental net benefit compared with a ‘treat all’ strategy (online supplemental efigure 4). In contrast, the refined and retrained models showed modest but consistent net benefits up to threshold probabilities of approximately 0.25 (online supplemental). If a CHR-P assessment were considered necessary above a predicted probability of CHR-P status of 0.20 in clinical samples (ie, not detecting a CHR-P individual is four times as harmful as an unnecessary assessment), these two models demonstrated slightly higher clinical utility compared with the external validation model and the ‘treat all’ strategy, with the refined model performing better than the retrained model.

In the general population scenario (online supplemental efigure 5), neither the externally validated nor the retrained PPS models showed any clinical utility, as their lines were indistinguishable from the ‘treat none’ and ‘treat all’ strategies. The refined model exhibited a modest advantage at extremely low thresholds but offered no net benefit at or above the general population prevalence threshold (0.0172), indicating limited practical utility in this context.

## Discussion

This study presents the external validation and refinement of the PPS model,^16^ a tool designed to summarise environmental and developmental exposures associated with psychosis and to support classification of CHR-P status. Using data from the large, international AMP SCZ consortium, we evaluated the generalisability of the original PPS model to a large, independent sample and refined the model using expanded predictor information. Three major findings emerged: (1) the PPS original model retained good performance in the new AMP SCZ cohort (external validation); (2) the refined version of the model, based on the results of Aymerich *et al*’s meta-analysis,^18^ showed higher internally cross-validated performance than the retrained original model, which provides the fairest benchmark for estimating the incremental value of predictor refinement; (3) the PPS scores derived from the refined model showed a weak association with subthreshold positive symptoms and socio-occupational functioning within the CHR-P group.

First, the external validation supported generalisability to AMP SCZ (C-Index=0.777), while also showing the need for recalibration, consistent with differences in case mix and predictor distributions between the two datasets (online supplemental eTable 2). The overall performance was lower than in the EU-GEI dataset,^16^ but this was expected and consistent with decreased model performance in external validation procedures due to context-dependent variations in predictor distributions and measurement properties.^20 21 24^

Second, refining the PPS model led to a significant improvement in its ability to detect CHR-P individuals relative to the retrained original model. The performance of the refined model was comparable to that of the original model in the EU-GEI dataset^16^ (C-Index ≈0.91). However, while performance deterioration in future external validations is expected, this is likely to be attenuated given the large sample size of the current study. Refining the PPS model highlights the added value of new predictors such as loneliness and increased granularity of other key predictors such as childhood trauma and parental mental illness. Loneliness, in particular, showed the largest positive coefficient for CHR-P classification, in line with prior systematic evidence.^25^ Similarly, emotional abuse and parental psychopathology were among the most influential predictors. Notably, the largest meta-analysis to date found that emotional abuse was the strongest contributor among trauma subtypes, associated with a more than threefold increase in the odds of psychosis.^26^ Having a parent affected by psychosis, on the other hand, was found linked to nearly sixfold higher odds of developing psychosis relative to a control offspring.^27^ For the interpretation of other coefficients, see online supplemental eDiscussion.

Third, and lastly, PPS scores were only weakly associated with greater subthreshold positive symptoms and poorer socio-occupational functioning. These findings suggest that, while accumulating environmental exposure may have measurable neurobiological consequences even before full psychosis onset,^11^ PPS-derived classification scores and subthreshold positive symptoms are related but distinct constructs. This supports the idea that PPS and symptom-based assessments (eg, CAARMS/SIPS) may complement each other within a stepped assessment framework, where the PPS represents a scalable, non-invasive measure of environmental and developmental exposures associated with CHR-P status, while symptom-centred interviews are reserved for individuals exceeding predefined classification thresholds. A similar framework is already being piloted in projects such as the E-DetectioN Tool for EaRly-stage at risk mental state in the community (ENTER) study.^28^ While ENTER is applied to the general population, other approaches may leverage the PPS by targeting other populations, including primary^29^ and secondary care.^30^ Alternatively, the PPS can be used to refine symptom-based prescreening measures such as the PQ-16.^14^

The use of decision curve analysis suggested that the PPS may have utility in enriched or help-seeking contexts, indicating that PPS-based classification could help streamline resource allocation by prioritising those most likely to meet CHR-P criteria. Conversely, none of the PPS models yielded clinical utility when assuming a general population context. While the CHR-P prevalence used (1.72%) might have been too low to enable meaningful clinical discrimination, these results emphasise the importance of prescreening within the general population. In this sense, a three-stage pathway based on symptom-centred prescreening tools such as the PQ-16, followed by a systematic investigation of environmental risk factors using the PPS, and a final semistructured interview (CAARMS/SIPS/PSYCHS) may represent an effective strategy. Whether the PPS could also be deployed for stratification within already-identified CHR-P samples remains instead an open question. Taken together, these findings do not support use of the PPS as a standalone tool for general-population screening. Rather, they suggest that any potential clinical utility is more plausible in enriched or help-seeking contexts, where CHR-P prevalence is expected to be higher than in the general population but lower than in highly enriched CHR-P clinical samples.

Despite its promising findings, the present study has some limitations to be acknowledged. First, the cross-sectional design does not permit establishing causal or temporal relationships between predictors and CHR-P status. Some predictors, including loneliness, anhedonia and social withdrawal, may also reflect features of the emerging prodromal state rather than antecedent environmental risk factors, raising the possibility of reverse causation. Second, the case-control design precludes inference about the absolute probability (or real-world incidence) of CHR-P status, as the proportion of cases and controls is deliberately fixed. Consequently, predicted probabilities derived from the model may be biased or inflated when applied to general population settings, where CHR-P status prevalence is substantially lower. The case-control design may also be susceptible to spectrum bias in that sampled individuals may differ markedly from those in true clinical settings, exaggerating the contrast between groups. In this regard, it is important to highlight that the control group of this study was a community sample with some individuals reporting past or current comorbidities including depression or passive suicidal ideation.^19^ As such, although the case-control design may exaggerate the distinction between groups in some respects, the inclusion of less stringently defined controls may have attenuated observed differences between CHR-P individuals and CC. The present findings, including sample-dependent metrics such as positive and negative predictive value (PPV/NPV), should be therefore interpreted as evidence of generalisability to the AMP SCZ validation context rather than definitive evidence of generalisability across all clinical, cultural or geographical settings. Third, several predictors relied on retrospective, self-reported measures and are therefore prone to recall bias. Fourth, not all theoretically relevant exposures could be included due to data availability (eg, urbanicity, ethnic density and pollution).^12 16^ Fifth, although methodologically conservative, the exclusion of predictors with missingness >20% (ie, paternal age and childhood social withdrawal) did not allow assessment of the potential incremental value of these variables, limiting the generalisability of the final predictor set to datasets in which paternal age or premorbid social withdrawal are measured. Future external validations should test whether these variables contribute additional predictive information when available with lower missingness, particularly given prior umbrella-review evidence reporting childhood social withdrawal and paternal age among risk factors for psychotic disorders.^12 18^ Sixth, the effects of certain environmental exposures (particularly ethnicity) may not be uniform across countries or sociocultural contexts. Globally developed models such as the refined PPS could benefit from local adaptation to maintain optimal performance. Lastly, the refined PPS model was internally validated using RN-CV but has not yet been externally validated in an independent cohort. External validation of the refined model in other datasets is therefore required before stronger claims regarding its broader generalisability can be made.

In conclusion, this study supports the generalisability of the original PPS model to a large, independent sample. Refined through the addition of new and more granular predictors, the PPS showed improved discrimination and calibration performances, although its clinical utility appears most plausible in enriched or help-seeking contexts rather than as a standalone general-population screening tool. Future work should externally validate the PPS refined model and assess its longitudinal utility in predicting transition to psychosis, along with its feasibility in stepped/sequential assessment frameworks for CHR-P detection and stratification across more diverse settings. In such pathways, use of the PPS would require trained clinical or research users to collect the relevant predictor data, apply the scoring algorithm and interpret the resulting score as part of a broader CHR-P assessment process.

## Supplementary material

10.1136/bmjment-2026-302838 online supplemental file 1

## Data Availability

Data may be obtained from a third party and are not publicly available.
