# Supplementary material for: External validation and refinement of the Psychosis Polyrisk Score to detect individuals at clinical high risk for psychosis
Source: BMJ Ment Health. 2026 Sep 28;29(1):e302838. doi: 10.1136/bmjment-2026-302838 (PMC13629901; doi:10.1136/bmjment-2026-302838)
Supplement: Supplementary file 1 [file bmjment-29-1-s001.docx]

**Supplementary Online Content**

**eTable 1.** List of the variables and the instrument used to derive each of them.

**eMethods.**

**eTable 2**. Comparison between the EU-GEI development dataset and AMP®SCZ validation dataset.

**eTable 3**. Performance metrics of the original PPS model (external validation, before recalibration).

**eFigure 1**. Calibration plot of the original PPS model (external validation).

**eTable 4.** PPS original model (external validation) – classification performances across thresholds.

**eTable 5.** Recalibrated PPS original model (external validation) – classification performances across thresholds.

**eTable 6.** Percentage of missing data in the external validation of the original PPS model.

**eTable 7**. Subgroup performance of the externally validated PPS model in AMP®SCZ by gender and ethnicity.

**eTable 8.** Per-fold percentage of missing data within refined PPS model.

**eTable 9.** Per-fold classification indices from the refined PPS model using RN-CV.

**eTable 10.** Per-fold calibration indices from the refined PPS model using RN-CV.

**eTable 11.** Per-fold classification indices from the retrained PPS model using RN-CV.

**eTable 12.** Per-fold calibration indices from the retrained PPS model using RN-CV.

**eTable 13.** Subgroup performance of the refined PPS model in AMP®SCZ by gender and ethnicity.

**eTable 14**. Leave-one-site-out cross validation.

**eTable 15**. Performance metrics of the refined PPS model excluding some predictors (sensitivity analyses).

**eFigure 2**. Calibration plots per each of the three PPS models.

**eTable 16**. Logistic regression coefficients of the refined PPS model.

**eTable 17.** Dropped or shrunk-to-zero variables within the refined PPS model.

**eTable 18**. Logistic regression coefficients of the original PPS model (used for the external validation) and its retrained version within the AMPSCZ dataset.

**eFigure 3.** Association of PPS scores with subthreshold positive symptoms and socio-occupational functioning restricted to CHR individuals.

**eFigure 4.** Decision curve analysis (DCA) comparing the three PPS models within the clinical population.

**eFigure 5.** Decision curve analysis (DCA) comparing the three PPS models within the general population

**eDiscussion**

**eReferences**

**eTable 1.** List of the variables and the instrument used to derive each of them.

| **Instrument** | **Variable** |
| --- | --- |
| Sociodemographic Questionnaire^a^ | age |
|  | gender |
|  | ethnicity |
|  | paternal age |
|  | handedness |
|  | first generation migrant |
|  | second generation migrant |
|  | North African first generation migrant |
|  | North African second generation migrant |
|  | parental socioeconomic status |
| Family Interview for Genetic Studies (FIGS)^1^ ^b^ | mother psychosis |
|  | mother other mental illness |
|  | father psychosis |
|  | father other mental illness |
| Life Threatening Events Questionnaire (LTE-Q)^2^ ^c^ | adult life events |
| Alcohol. Smoking. and Substance Involvement Screening Test (ASSIST)^3^ ^d^ | tobacco daily |
|  | cannabis daily |
| Adapted version of the Childhood Trauma Questionnaire-Short Form (CTQ-SF)^4^ ^e^ | childhood trauma (total) |
|  | emotional abuse |
|  | physical abuse |
|  | sexual abuse |
|  | emotional neglect |
|  | physical neglect |
| The 6-item De Jong Gierveld Loneliness Scale^5^ ^f^ | loneliness |
| Merging the "social disorganization (SocD)" and "social cohesion and trust (SC&T)" items^6^ ^g^ | neighborhood quality |
| Temporal Experience of Pleasure Scale (TEPS)^7^ ^h^ | anhedonia |
| Wide Range Achievement Test – Fifth Edition (WRAT-5)^8^ ^i^ | premorbid IQ |
| Premorbid Adjustment Scale (PAS)^9^ ^l^ | childhood social withdrawal |

Below a summary of how predictors were coded:

^a^ For all sociodemographics, categorical scores were used (1 = present, 0 = absent).

^b^ For family history of psychopathology, categorical scores were used (1 = diagnosis certainly present or probable, 0 = no diagnosis). Psychosis includes schizophrenia, schizoaffective disorder and other non-affective disorders. Other mental illness includes major depressive disorder, bipolar disorder, suicidality.

^c^ For adult life events, categorical scores were used (1 = at least one stressful life event occurred during the past six months, 0 = no stressful life events during the past six months).

^d^ For tobacco and cannabis use, categorical scores were used (1= daily tobacco/cannabis, 0 = no daily tobacco/cannabis)

^e^ For childhood trauma total score, a cutoff of 55 was used to establish whether a participant experienced childhood or not in the original PPS model (predictor set 1). Conversely, in the refined PPS model (predictor set 2), childhood trauma domains were considered as continuous predictors.

^f^ For loneliness, continuous scores were used.

^g^ For neighborhood quality, continuous scores were used.

^h^ For anhedonia total score, a cutoff of 35 was used to establish whether a participant experiences anhedonia or not in the original PPS model (predictor set 1). Conversely, in the refined PPS model (predictor set 2), anhedonia was considered as a continuous predictor.

^i^ For premorbid IQ, continuous scores were used.

^l^ For childhood social withdrawal a continuous score was used, and taken from the item “Sociability and Withdrawal” of the Premorbid Adjustment Scale (PAS).

**eMethods.**

A post hoc sample-size adequacy calculation^10^ using the R package pmsampsize version 1.1.3 indicated that, assuming 32 candidate predictor parameters from predictor set 2, a C-statistic of 0.88 and the outcome proportion of 0.837 observed in the EU-GEI PPS development study^11^, the minimum required sample size was 919 participants with 770 CHR cases. The available AMP^®^SCZ sample used for model refinement included 2161 participants, including 1642 CHR individuals, exceeding this requirement.

CHR status was determined by researchers and clinicians by the presence of at least one of the following criteria:

i. Attenuated psychotic symptoms (APS) evaluated using the Positive Symptoms and Diagnostic Criteria for the Comprehensive Assessment of At-Risk Mental States (CAARMS^12^), harmonized with the Structured Interview for Psychosis Risk Syndromes (SIPS^13^) (PSYCHS)^14^;
ii. Brief limited intermittent psychotic symptoms (BLIPS) evaluated using the PSYCHS^14^;
iii. Trait vulnerability and genetic risk with functional deterioration, assessed using the schizotypal personality disorder section from the Structured Clinical Interview for DSM-5^15^, the Social and Occupational Functioning Assessment Scale (SOFAS)^16^, and the Family Interview for Genetic Studies (FIGS)^1^.

CHR participants were excluded if they had a history of antipsychotic medication exposure exceeding a haloperidol-equivalent lifetime dose of 50 mg, a clinically relevant central nervous system disorder, or a traumatic brain injury rated ≥7 on the Traumatic Brain Injury Screening Instrument.

CC were recruited from the community and matched to CHR participants on age, sex, and educational background. They were excluded if they had a history of psychotic disorders, any CHR psychosis-risk syndrome, Cluster A personality disorder, documented intellectual disability, current or past central nervous system disorder, traumatic brain injury (≥ 7), current psychotropic medication use, or a first-degree relative with a full-blown psychotic spectrum disorder.

**eTable 2**. Comparison between the EU-GEI development dataset and AMP®SCZ validation dataset

| **Domain** | **EU-GEI development dataset** | **AMP®SCZ validation dataset** | **Conclusions** |
| --- | --- | --- | --- |
| Study role | Development and internal validation dataset for the original PPS model. | External validation dataset for the original PPS model (also used for retraining and refinement analyses). | Clarifies that the original PPS coefficients were estimated in EU-GEI and externally tested in AMP®SCZ . |
| Study design | International, multicentre prospective cohort study. | International, multisite cohort from the AMP®SCZ program, including ProNET and PRESCIENT networks. | Both are multisite CHR-P studies, but AMP®SCZ is larger and more globally distributed. |
| Recruitment period | May 2010 to April 2015. | 2020 (no same start for each site) to February 2025 | The AMP®SCZ dataset was collected approximately five to fifteen years after the EU-GEI development dataset, so temporal differences in recruitment period may have contributed to differences in case mix, referral pathways, assessment practices, and exposure distributions between the two samples. |
| Geographical coverage | Amsterdam, Barcelona, Basel, Cologne, Copenhagen, Den Haag, London, Paris, Vienna, Melbourne, and São Paulo. Healthy controls were recruited from four sites: Amsterdam, Den Haag, London, and Melbourne. | Singapore; Hong Kong; Cologne, Germany; Santiago, Chile; USA; Montreal, Canada; King’s College London, UK; Pavia, Italy; Madrid, Spain; Cambridge, UK; Ludwig Maximilian University Munich, Germany; Seoul, Korea; Ohio State University, USA; University of Rochester, USA; Melbourne, Australia; Jena, Germany; Copenhagen, Denmark; Birmingham, UK; Lausanne, Switzerland; Gwangju, Korea. | AMP®SCZ provides a broader and more heterogeneous external validation context. |
| Sample size for CHR-P detection/classification | N = 344 CHR-P; N = 67 healthy controls. | N = 1642 CHR; N = 519 community controls. | AMP®SCZ is substantially larger, increasing precision but also introducing broader case-mix heterogeneity. |
| Comparator group | Healthy controls. | Community controls. | Although labelled “healthy”, controls in EU-GEI also had mental comorbidities, thus comparable with the AMP®SCZ community controls. For demographics differences see below. |
| CHR-P definition | CHR-P status assessed using CAARMS. | CHR-P status assessed using PSYCHS criteria harmonising CAARMS and SIPS information. | Outcome constructs are closely related but not identical in operationalisation. |
| Modelled outcome | Baseline CHR-P vs healthy-control status. | Baseline CHR-P vs community-control status. | Both analyses model concurrent CHR-P classification (not prospective transition to psychosis). |
| Main exclusion criteria for CHR-P participants | Previous psychotic disorder, exceeding CAARMS psychosis/antipsychotic threshold, IQ < 60, unwillingness to provide genetic sample. | Antipsychotic exposure exceeding 50 mg haloperidol-equivalent lifetime dose, clinically relevant central nervous system disorder, or traumatic brain injury rated ≥ 7. | Eligibility criteria slightly differ and may contribute to case-mix differences. |
| Main exclusion criteria for controls | Healthy controls were excluded if they met CAARMS criteria for CHR-P. | Community controls were excluded if they had psychotic disorder history, any CHR syndrome, Cluster A personality disorder, documented intellectual disability, current/past CNS disorder, traumatic brain injury ≥ 7, current psychotropic medication use, or a first-degree relative with a psychotic spectrum disorder. | AMP®SCZ control criteria were stricter for some domains, including family history of psychosis. |
| Age | Healthy controls: 22.9 ± 4.1; CHR-P: 22.4 ± 4.9. | Community controls: 21.6 ± 3.6; CHR: 21.2 ± 4.0. | Age distributions were broadly comparable. |
| Gender | Male: healthy controls 50.7%; CHR-P 53.8%. | Female: community controls 60.9%; CHR 54.8%. Male: community controls 37.5%; CHR 35.2%. Non-binary: community controls 1.6%; CHR 8.2%. Other gender: community controls 0%; CHR 1.8%. | EU-GEI used binary gender coding, whereas AMP®SCZ included more granular gender categories. For the external validation, the AMP®SCZ dataset was restricted to participants whose recorded gender was female or male to match the coding of the original EU-GEI PPS model. |
| Ethnicity | White: healthy controls 62.7%; CHR-P 71.8%. Black: healthy controls 14.9%; CHR-P 9.9%. Other: healthy controls 22.4%; CHR-P 18.3%. | White: healthy controls 51.8%; CHR-P 58.5%. Black: healthy controls 6.2%; CHR-P 8.3%. Other: healthy controls 41.0%; CHR-P 33.2%. | White and black ethnicity was more prevalent in EU-GEI participants, whereas Other ethnicity was higher in AMP®SCZ due to the broad geographical coverage. |
| Migration status | First-generation immigrant: healthy controls 28.4%; CHR-P 24.4%. Second-generation immigrant: healthy controls 25.4%; CHR-P 26.2%. First-generation North African immigrant: healthy controls 0%; CHR-P 0.3%. Second-generation North African immigrant: healthy controls 0%; CHR-P 1.5%. | First-generation immigrant: community controls 22.5%; CHR 14.8%. Second-generation immigrant: community controls 17.0%; CHR 16.4%. First-generation North African immigrant: community controls 0.2%; CHR 0.1%. Second-generation North African immigrant: community controls 0.4%; CHR 0.2%. | Distributions differed across the two datasets, especially regarding first- and second-generation migrants |
| Low parental socioeconomic status | Healthy controls 12.1%; CHR-P 19.1%. | Community controls 5.9%; CHR-P 16.0%. | Broadly comparable distributions, though paternal socioeconomic status was not available in AMP®SCZ (parental socioeconomic status was used). |
| Parental severe mental illness | Healthy controls 27.5%; CHR-P 64.2%. | Community controls 14.8%; CHR-P 59.5%. | More prevalent in EU-GEI healthy controls than in AMP®SCZ community controls (similar distribution in CHR-P) |
| Adult stressful life events | Healthy controls 68.7%; CHR-P 85.8%. | Community controls 44.5%; CHR-P 74.2%. | Higher rates in CHR-P groups in both studies, but more stressful life events in EU-GEI controls. |
| Tobacco use (daily) | Healthy controls 25.4%; CHR-P 51.0%. | Community controls 5.7%; CHR-P 13.8%. | Distributions differed substantially. Possibly due to recruitment or measurement differences. Also, smoking has become less prevalent over time |
| Cannabis use | Heavy cannabis use: healthy controls 13.4%; CHR-P 33.2%. | Heavy cannabis use: community controls 8.3%; CHR-P 17.9%. | Heavy cannabis use (i.e., cannabis use more than once a week or daily) was larger in the EU-GEI CHR-P population. |
| Childhood trauma (i.e., total score > 55) | Healthy controls 3.0%; CHR-P 26.3%. | Community controls 5.8%. CHR-P 33.9%. | Comparable distributions across the two datasets. |
| Trait anhedonia (i.e., total score > 35) | Healthy controls 12.1%; CHR-P 62.3%. | Healthy controls 16%; CHR-P 33.6%. | Comparable distributions in controls; more anhedonia levels in the EU-GEI CHR-P participants. |
| Non-right handedness | Healthy controls 9.8%; CHR-P 10.8%. | Community controls 7.5%; CHR-P 9.9%. | Broadly comparable distribution. |
| High urbanicity | Healthy controls 98.5%; CHR-P 95.9%. | Not yet available in the present AMP®SCZ predictor set. | This original PPS predictor could not be externally validated/refined in AMP®SCZ. |
| High pollution | Healthy controls 100.0%; CHR-P 90.1%. | Not yet available in the present AMP®SCZ predictor set. | This original PPS predictor could not be externally validated/refined in AMP®SCZ. |
| High ethnic density | Healthy controls 84.6%; CHR-P 50.2%. Low ethnic density: healthy controls 13.8%; CHR-P 44.4%. | Not yet available in the present AMP®SCZ predictor set. | This original PPS predictor could not be externally validated/refined in AMP®SCZ. |
| Data missingness considerations | Missing data were imputed following investigation of missingness patterns using the naniar package and imputed through Random forest. imputation | Variables with >20% missingness were excluded. Remaining missing variables were imputed using random forest imputation. | Missingness patterns differed across datasets, but the imputation process was similar. |
| Participant flow | EU-GEI included 344 CHR-P and 67 healthy controls in the detection analysis. | AMP®SCZ Release 3.0 included 1642 CHR and 519 community controls. Exact screening and exclusion flow numbers are not currently available. | AMP®SCZ is larger sample, but exact recruitment numbers are not currently available due to the still ongoing data cleaning and formatting |

**eTable 3**. Performance metrics of the original PPS model (external validation, before recalibration).

|  | **Estimate** | **Lower 95CI** | **Upper 95CI** |
| --- | --- | --- | --- |
| **Balanced Accuracy** | 0.705698569 | 0.68521928 | 0.725029747 |
| **Sensitivity** | 0.563742996 | 0.538367844 | 0.587088916 |
| **Specificity** | 0.847654143 | 0.815077463 | 0.878612717 |
| **PPV** | 0.921335298 | 0.905834831 | 0.937069461 |
| **NPV** | 0.380562113 | 0.364807764 | 0.396092362 |
| **Brier Score** | 0.168716718 | 0.163379431 | 0.173928461 |
| **Calibration Intercept** | -0.968239255 | -1.020067244 | -0.916770771 |
| **Calibration Slope** | 0.942579189 | 0.836118504 | 1.05999769 |
| **ICI** | 0.260229879 | 0.255017294 | 0.265640627 |
| **Harrell's C** | 0.77592186 | 0.754098275 | 0.797300446 |

**eFigure 1**. Calibration plot of the of the original PPS model (external validation).

**eTable 4.** PPS original model (external validation) – classification performances across thresholds*.

| **Threshold** | **BAC** | **Sensitivity** | **Specificity** | **PPV** | **NPV** |
| --- | --- | --- | --- | --- | --- |
| 0 | 0.5 | 1 | 0 | 0.75983341 | - |
| 0.01 | 0.5 | 1 | 0 | 0.75983341 | - |
| 0.02 | 0.5 | 1 | 0 | 0.75983341 | - |
| 0.03 | 0.5 | 1 | 0 | 0.75983341 | - |
| 0.04 | 0.5 | 1 | 0 | 0.75983341 | - |
| 0.05 | 0.5 | 1 | 0 | 0.75983341 | - |
| 0.06 | 0.5 | 1 | 0 | 0.75983341 | - |
| 0.07 | 0.5 | 1 | 0 | 0.75983341 | - |
| 0.08 | 0.5 | 1 | 0 | 0.75983341 | - |
| 0.09 | 0.5 | 1 | 0 | 0.75983341 | - |
| 0.1 | 0.5 | 1 | 0 | 0.75983341 | - |
| 0.11 | 0.5 | 1 | 0 | 0.75983341 | - |
| 0.12 | 0.5 | 1 | 0 | 0.75983341 | - |
| 0.13 | 0.5 | 1 | 0 | 0.75983341 | - |
| 0.14 | 0.5 | 1 | 0 | 0.75983341 | - |
| 0.15 | 0.5 | 1 | 0 | 0.75983341 | - |
| 0.16 | 0.5 | 1 | 0 | 0.75983341 | - |
| 0.17 | 0.5 | 1 | 0 | 0.75983341 | - |
| 0.18 | 0.5 | 1 | 0 | 0.75983341 | - |
| 0.19 | 0.5 | 1 | 0 | 0.75983341 | - |
| 0.2 | 0.5 | 1 | 0 | 0.75983341 | - |
| 0.21 | 0.5 | 1 | 0 | 0.75983341 | - |
| 0.22 | 0.5 | 1 | 0 | 0.75983341 | - |
| 0.23 | 0.5 | 1 | 0 | 0.75983341 | - |
| 0.24 | 0.5 | 1 | 0 | 0.75983341 | - |
| 0.25 | 0.5 | 1 | 0 | 0.75983341 | - |
| 0.26 | 0.5 | 1 | 0 | 0.75983341 | - |
| 0.27 | 0.5 | 1 | 0 | 0.75983341 | - |
| 0.28 | 0.5 | 1 | 0 | 0.75983341 | - |
| 0.29 | 0.50836601 | 0.99939099 | 0.01734104 | 0.76290098 | 0.9 |
| 0.3 | 0.5090249 | 0.99878197 | 0.01926782 | 0.76314565 | 0.83333333 |
| 0.31 | 0.51262383 | 0.99634592 | 0.02890173 | 0.76448598 | 0.71428571 |
| 0.32 | 0.51358722 | 0.99634592 | 0.03082852 | 0.76484338 | 0.72727273 |
| 0.33 | 0.51683177 | 0.99512789 | 0.03853565 | 0.7660572 | 0.71428571 |
| 0.34 | 0.51683177 | 0.99512789 | 0.03853565 | 0.7660572 | 0.71428571 |
| 0.35 | 0.51779516 | 0.99512789 | 0.04046243 | 0.76641651 | 0.72413793 |
| 0.36 | 0.51779516 | 0.99512789 | 0.04046243 | 0.76641651 | 0.72413793 |
| 0.37 | 0.51779516 | 0.99512789 | 0.04046243 | 0.76641651 | 0.72413793 |
| 0.38 | 0.51911293 | 0.99390987 | 0.04431599 | 0.76691729 | 0.6969697 |
| 0.39 | 0.52007632 | 0.99390987 | 0.04624277 | 0.76727786 | 0.70588235 |
| 0.4 | 0.52590654 | 0.99208283 | 0.05973025 | 0.76948512 | 0.70454545 |
| 0.41 | 0.52783332 | 0.99208283 | 0.06358382 | 0.77021277 | 0.7173913 |
| 0.42 | 0.53138238 | 0.99147381 | 0.07129094 | 0.77156398 | 0.7254902 |
| 0.43 | 0.53138238 | 0.99147381 | 0.07129094 | 0.77156398 | 0.7254902 |
| 0.44 | 0.54750539 | 0.98903776 | 0.10597303 | 0.77777778 | 0.75342466 |
| 0.45 | 0.55394462 | 0.98842875 | 0.1194605 | 0.78028846 | 0.7654321 |
| 0.46 | 0.55490801 | 0.98842875 | 0.12138728 | 0.78066378 | 0.76829268 |
| 0.47 | 0.5568348 | 0.98842875 | 0.12524085 | 0.7814155 | 0.77380952 |
| 0.48 | 0.5568348 | 0.98842875 | 0.12524085 | 0.7814155 | 0.77380952 |
| 0.49 | 0.55749368 | 0.98781973 | 0.12716763 | 0.78168675 | 0.76744186 |
| 0.5 | 0.55749368 | 0.98781973 | 0.12716763 | 0.78168675 | 0.76744186 |
| 0.51 | 0.55881145 | 0.98660171 | 0.13102119 | 0.78223081 | 0.75555556 |
| 0.52 | 0.55881145 | 0.98660171 | 0.13102119 | 0.78223081 | 0.75555556 |
| 0.53 | 0.55881145 | 0.98660171 | 0.13102119 | 0.78223081 | 0.75555556 |
| 0.54 | 0.56504592 | 0.9817296 | 0.14836224 | 0.78481013 | 0.71962617 |
| 0.55 | 0.56732708 | 0.98051157 | 0.15414258 | 0.78574915 | 0.71428571 |
| 0.56 | 0.56732708 | 0.98051157 | 0.15414258 | 0.78574915 | 0.71428571 |
| 0.57 | 0.56550004 | 0.97685749 | 0.15414258 | 0.78511992 | 0.6779661 |
| 0.58 | 0.56742682 | 0.97685749 | 0.15799615 | 0.78588927 | 0.68333333 |
| 0.59 | 0.56717218 | 0.97442144 | 0.15992293 | 0.78585462 | 0.664 |
| 0.6 | 0.56909897 | 0.97442144 | 0.16377649 | 0.78662734 | 0.66929134 |
| 0.61 | 0.5717345 | 0.97198538 | 0.17148362 | 0.78775913 | 0.65925926 |
| 0.62 | 0.57239339 | 0.97137637 | 0.1734104 | 0.78804348 | 0.65693431 |
| 0.63 | 0.57122523 | 0.96711328 | 0.17533719 | 0.78769841 | 0.62758621 |
| 0.64 | 0.57320189 | 0.96528624 | 0.18111753 | 0.78855721 | 0.62251656 |
| 0.65 | 0.57416528 | 0.96528624 | 0.18304432 | 0.78894973 | 0.625 |
| 0.66 | 0.57386077 | 0.96467722 | 0.18304432 | 0.78884462 | 0.62091503 |
| 0.67 | 0.57386077 | 0.96467722 | 0.18304432 | 0.78884462 | 0.62091503 |
| 0.68 | 0.57421515 | 0.9634592 | 0.1849711 | 0.78902743 | 0.61538462 |
| 0.69 | 0.57487403 | 0.96285018 | 0.18689788 | 0.78931603 | 0.61392405 |
| 0.7 | 0.57583742 | 0.96285018 | 0.18882466 | 0.78971029 | 0.6163522 |
| 0.71 | 0.57649631 | 0.96224117 | 0.19075145 | 0.79 | 0.61490683 |
| 0.72 | 0.57770905 | 0.95310597 | 0.20231214 | 0.79080344 | 0.57692308 |
| 0.73 | 0.57841781 | 0.95066991 | 0.2061657 | 0.79118094 | 0.56914894 |
| 0.74 | 0.5778088 | 0.94945189 | 0.2061657 | 0.79096905 | 0.56315789 |
| 0.75 | 0.59722623 | 0.94397077 | 0.2504817 | 0.79938112 | 0.58558559 |
| 0.76 | 0.5998119 | 0.94336175 | 0.25626204 | 0.8005168 | 0.58849558 |
| 0.77 | 0.6196283 | 0.92326431 | 0.31599229 | 0.81026189 | 0.56551724 |
| 0.78 | 0.62895243 | 0.91108404 | 0.34682081 | 0.81525886 | 0.55214724 |
| 0.79 | 0.62864792 | 0.91047503 | 0.34682081 | 0.81515812 | 0.55045872 |
| 0.8 | 0.62869779 | 0.90864799 | 0.34874759 | 0.81530055 | 0.54682779 |
| 0.81 | 0.62971105 | 0.90682095 | 0.35260116 | 0.81589041 | 0.54464286 |
| 0.82 | 0.63654397 | 0.88002436 | 0.39306358 | 0.82102273 | 0.50872818 |
| 0.83 | 0.63946818 | 0.84348356 | 0.43545279 | 0.82538737 | 0.4679089 |
| 0.84 | 0.65842093 | 0.81973203 | 0.49710983 | 0.83758556 | 0.46570397 |
| 0.85 | 0.69379593 | 0.78258222 | 0.60500963 | 0.86241611 | 0.46795827 |
| 0.86 | 0.69378536 | 0.75943971 | 0.62813102 | 0.86597222 | 0.45214979 |
| 0.87 | 0.69631588 | 0.74908648 | 0.64354528 | 0.86925795 | 0.44772118 |
| 0.88 | 0.69656523 | 0.73995128 | 0.65317919 | 0.87096774 | 0.44255875 |
| 0.89 | 0.69797747 | 0.72350792 | 0.67244701 | 0.87481591 | 0.43462017 |
| 0.9 | 0.71273519 | 0.6181486 | 0.80732177 | 0.9103139 | 0.40057361 |
| 0.91 | 0.70527213 | 0.59744214 | 0.81310212 | 0.91001855 | 0.38965836 |
| 0.92 | 0.7058653 | 0.56394641 | 0.8477842 | 0.92139303 | 0.38062284 |
| 0.93 | 0.6985413 | 0.51461632 | 0.88246628 | 0.93267108 | 0.36494024 |
| 0.94 | 0.691787 | 0.49147381 | 0.89210019 | 0.93511008 | 0.35670262 |
| 0.95 | 0.66299264 | 0.39342266 | 0.93256262 | 0.94860499 | 0.32702703 |
| 0.96 | 0.64415253 | 0.33069428 | 0.95761079 | 0.96106195 | 0.31140351 |
| 0.97 | 0.63470226 | 0.28867235 | 0.98073218 | 0.97933884 | 0.30351819 |
| 0.98 | 0.59186656 | 0.19914738 | 0.98458574 | 0.9761194 | 0.27984666 |
| 0.99 | 0.53217797 | 0.0682095 | 0.99614644 | 0.98245614 | 0.25256473 |
| 1 | 0.5 | 0 | 1 | - | 0.24016659 |

*before recalibration

**eTable 5.** Recalibrated PPS original model (external validation) – classification performances across thresholds.

| **Threshold** | **BAC** | **Sensitivity** | **Specificity** | **PPV** | **NPV** |
| --- | --- | --- | --- | --- | --- |
| 0 | 0.5 | 1 | 0 | 0.75983341 | - |
| 0.01 | 0.5 | 1 | 0 | 0.75983341 | - |
| 0.02 | 0.5 | 1 | 0 | 0.75983341 | - |
| 0.03 | 0.5 | 1 | 0 | 0.75983341 | - |
| 0.04 | 0.5 | 1 | 0 | 0.75983341 | - |
| 0.05 | 0.5 | 1 | 0 | 0.75983341 | - |
| 0.06 | 0.5 | 1 | 0 | 0.75983341 | - |
| 0.07 | 0.5 | 1 | 0 | 0.75983341 | - |
| 0.08 | 0.5 | 1 | 0 | 0.75983341 | - |
| 0.09 | 0.5 | 1 | 0 | 0.75983341 | - |
| 0.1 | 0.5 | 1 | 0 | 0.75983341 | - |
| 0.11 | 0.5 | 1 | 0 | 0.75983341 | - |
| 0.12 | 0.5 | 1 | 0 | 0.75983341 | - |
| 0.13 | 0.5 | 1 | 0 | 0.75983341 | - |
| 0.14 | 0.5 | 1 | 0 | 0.75983341 | - |
| 0.15 | 0.5 | 1 | 0 | 0.75983341 | - |
| 0.16 | 0.50836601 | 0.99939099 | 0.01734104 | 0.76290098 | 0.9 |
| 0.17 | 0.51262383 | 0.99634592 | 0.02890173 | 0.76448598 | 0.71428571 |
| 0.18 | 0.51683177 | 0.99512789 | 0.03853565 | 0.7660572 | 0.71428571 |
| 0.19 | 0.51779516 | 0.99512789 | 0.04046243 | 0.76641651 | 0.72413793 |
| 0.2 | 0.51779516 | 0.99512789 | 0.04046243 | 0.76641651 | 0.72413793 |
| 0.21 | 0.51911293 | 0.99390987 | 0.04431599 | 0.76691729 | 0.6969697 |
| 0.22 | 0.52007632 | 0.99390987 | 0.04624277 | 0.76727786 | 0.70588235 |
| 0.23 | 0.52686993 | 0.99208283 | 0.06165703 | 0.76984877 | 0.71111111 |
| 0.24 | 0.53138238 | 0.99147381 | 0.07129094 | 0.77156398 | 0.7254902 |
| 0.25 | 0.54750539 | 0.98903776 | 0.10597303 | 0.77777778 | 0.75342466 |
| 0.26 | 0.54846878 | 0.98903776 | 0.10789981 | 0.77815046 | 0.75675676 |
| 0.27 | 0.55490801 | 0.98842875 | 0.12138728 | 0.78066378 | 0.76829268 |
| 0.28 | 0.5568348 | 0.98842875 | 0.12524085 | 0.7814155 | 0.77380952 |
| 0.29 | 0.55749368 | 0.98781973 | 0.12716763 | 0.78168675 | 0.76744186 |
| 0.3 | 0.55749368 | 0.98781973 | 0.12716763 | 0.78168675 | 0.76744186 |
| 0.31 | 0.55881145 | 0.98660171 | 0.13102119 | 0.78223081 | 0.75555556 |
| 0.32 | 0.55881145 | 0.98660171 | 0.13102119 | 0.78223081 | 0.75555556 |
| 0.33 | 0.56504592 | 0.9817296 | 0.14836224 | 0.78481013 | 0.71962617 |
| 0.34 | 0.56732708 | 0.98051157 | 0.15414258 | 0.78574915 | 0.71428571 |
| 0.35 | 0.56702257 | 0.97990256 | 0.15414258 | 0.78564453 | 0.7079646 |
| 0.36 | 0.56550004 | 0.97685749 | 0.15414258 | 0.78511992 | 0.6779661 |
| 0.37 | 0.56742682 | 0.97685749 | 0.15799615 | 0.78588927 | 0.68333333 |
| 0.38 | 0.56717218 | 0.97442144 | 0.15992293 | 0.78585462 | 0.664 |
| 0.39 | 0.57011223 | 0.9725944 | 0.16763006 | 0.78708724 | 0.65909091 |
| 0.4 | 0.57239339 | 0.97137637 | 0.1734104 | 0.78804348 | 0.65693431 |
| 0.41 | 0.57122523 | 0.96711328 | 0.17533719 | 0.78769841 | 0.62758621 |
| 0.42 | 0.57320189 | 0.96528624 | 0.18111753 | 0.78855721 | 0.62251656 |
| 0.43 | 0.57416528 | 0.96528624 | 0.18304432 | 0.78894973 | 0.625 |
| 0.44 | 0.57386077 | 0.96467722 | 0.18304432 | 0.78884462 | 0.62091503 |
| 0.45 | 0.57386077 | 0.96467722 | 0.18304432 | 0.78884462 | 0.62091503 |
| 0.46 | 0.57386077 | 0.96467722 | 0.18304432 | 0.78884462 | 0.62091503 |
| 0.47 | 0.57421515 | 0.9634592 | 0.1849711 | 0.78902743 | 0.61538462 |
| 0.48 | 0.57583742 | 0.96285018 | 0.18882466 | 0.78971029 | 0.6163522 |
| 0.49 | 0.57553292 | 0.96224117 | 0.18882466 | 0.7896052 | 0.6125 |
| 0.5 | 0.57801356 | 0.95371498 | 0.20231214 | 0.79090909 | 0.5801105 |
| 0.51 | 0.57770905 | 0.95310597 | 0.20231214 | 0.79080344 | 0.57692308 |
| 0.52 | 0.57841781 | 0.95066991 | 0.2061657 | 0.79118094 | 0.56914894 |
| 0.53 | 0.5781133 | 0.9500609 | 0.2061657 | 0.79107505 | 0.56613757 |
| 0.54 | 0.59626284 | 0.94397077 | 0.24855491 | 0.79896907 | 0.58371041 |
| 0.55 | 0.59722623 | 0.94397077 | 0.2504817 | 0.79938112 | 0.58558559 |
| 0.56 | 0.5998119 | 0.94336175 | 0.25626204 | 0.8005168 | 0.58849558 |
| 0.57 | 0.6196283 | 0.92326431 | 0.31599229 | 0.81026189 | 0.56551724 |
| 0.58 | 0.62895243 | 0.91108404 | 0.34682081 | 0.81525886 | 0.55214724 |
| 0.59 | 0.62864792 | 0.91047503 | 0.34682081 | 0.81515812 | 0.55045872 |
| 0.6 | 0.62834341 | 0.90986602 | 0.34682081 | 0.81505728 | 0.54878049 |
| 0.61 | 0.62869779 | 0.90864799 | 0.34874759 | 0.81530055 | 0.54682779 |
| 0.62 | 0.63001556 | 0.90742996 | 0.35260116 | 0.81599124 | 0.54626866 |
| 0.63 | 0.62940655 | 0.90621194 | 0.35260116 | 0.81578947 | 0.54302671 |
| 0.64 | 0.63654397 | 0.88002436 | 0.39306358 | 0.82102273 | 0.50872818 |
| 0.65 | 0.63441243 | 0.87576127 | 0.39306358 | 0.82030804 | 0.5 |
| 0.66 | 0.64078594 | 0.84226553 | 0.43930636 | 0.82616487 | 0.46817248 |
| 0.67 | 0.65842093 | 0.81973203 | 0.49710983 | 0.83758556 | 0.46570397 |
| 0.68 | 0.65978857 | 0.81668697 | 0.50289017 | 0.83864916 | 0.46441281 |
| 0.69 | 0.68902356 | 0.76918392 | 0.6088632 | 0.86152797 | 0.45467626 |
| 0.7 | 0.69378536 | 0.75943971 | 0.62813102 | 0.86597222 | 0.45214979 |
| 0.71 | 0.695657 | 0.74969549 | 0.6416185 | 0.86873677 | 0.44758065 |
| 0.72 | 0.69575674 | 0.74604141 | 0.64547206 | 0.86941093 | 0.44547872 |
| 0.73 | 0.69656523 | 0.73995128 | 0.65317919 | 0.87096774 | 0.44255875 |
| 0.74 | 0.69874137 | 0.73081608 | 0.66666667 | 0.87399854 | 0.43908629 |
| 0.75 | 0.6982321 | 0.72594397 | 0.67052023 | 0.87454145 | 0.43609023 |
| 0.76 | 0.70955107 | 0.6406821 | 0.77842004 | 0.90145673 | 0.40643863 |
| 0.77 | 0.71385347 | 0.62423873 | 0.80346821 | 0.90949423 | 0.4032882 |
| 0.78 | 0.71065351 | 0.61205847 | 0.80924855 | 0.91032609 | 0.39735099 |
| 0.79 | 0.7040541 | 0.59500609 | 0.81310212 | 0.90968343 | 0.38822447 |
| 0.8 | 0.71150132 | 0.58099878 | 0.84200385 | 0.92084942 | 0.38844444 |
| 0.81 | 0.70561067 | 0.56151035 | 0.84971098 | 0.922 | 0.37984496 |
| 0.82 | 0.7035683 | 0.53045067 | 0.87668593 | 0.9315508 | 0.37112561 |
| 0.83 | 0.6985413 | 0.51461632 | 0.88246628 | 0.93267108 | 0.36494024 |
| 0.84 | 0.69686387 | 0.50548112 | 0.88824663 | 0.93468468 | 0.36213668 |
| 0.85 | 0.691787 | 0.49147381 | 0.89210019 | 0.93511008 | 0.35670262 |
| 0.86 | 0.69132232 | 0.47320341 | 0.90944123 | 0.94296117 | 0.35302917 |
| 0.87 | 0.66329714 | 0.39403167 | 0.93256262 | 0.94868035 | 0.32724814 |
| 0.88 | 0.65263413 | 0.36114495 | 0.94412331 | 0.95337621 | 0.31838856 |
| 0.89 | 0.64648884 | 0.33922046 | 0.95375723 | 0.95869191 | 0.31329114 |
| 0.9 | 0.63856639 | 0.31181486 | 0.96531792 | 0.96603774 | 0.30717351 |
| 0.91 | 0.63389377 | 0.29476248 | 0.97302505 | 0.97188755 | 0.30366807 |
| 0.92 | 0.63505664 | 0.28745432 | 0.98265896 | 0.98128898 | 0.30357143 |
| 0.93 | 0.605824 | 0.22898904 | 0.98265896 | 0.97662338 | 0.28716216 |
| 0.94 | 0.59856571 | 0.21254568 | 0.98458574 | 0.97759104 | 0.28325942 |
| 0.95 | 0.57734998 | 0.16626066 | 0.98843931 | 0.97849462 | 0.27258236 |
| 0.96 | 0.56278353 | 0.13520097 | 0.99036609 | 0.97797357 | 0.26577042 |
| 0.97 | 0.53217797 | 0.0682095 | 0.99614644 | 0.98245614 | 0.25256473 |
| 0.98 | 0.51887942 | 0.03775883 | 1 | 1 | 0.2472606 |
| 0.99 | 0.50091352 | 0.00182704 | 1 | 1 | 0.24050046 |
| 1 | 0.5 | 0 | 1 | - | 0.24016659 |

**eTable 6.** Percentage of missing data in the external validation of the original PPS model.

| **Predictor** | **Missingness (%)** |
| --- | --- |
| childhood trauma | 14.9467839 |
| anhedonia | 14.9467839 |
| parent smi | 12.49421564 |
| adult life events | 11.52244331 |
| tobacco daily | 6.432207311 |
| heavy cannabis use | 6.432207311 |
| age | 3.748264692 |
| parental socioeconomic status | 3.146691347 |
| ethnicity | 2.915316983 |
| gender | 1.573345673 |
| first generation migrant | 1.156871819 |
| second generation migrant | 1.156871819 |
| North African first generation migrant | 0 |
| North African second generation migrant | 0 |

**eTable 7**. Subgroup performance of the externally validated PPS model in AMP®SCZ by gender and ethnicity.

| **Model** | **Subgroup_variable** | **Subgroup** | **N** | **N_CHR** | **N_CC** | **Metric** | **Estimate** | **Lower_95CI** | **Upper_95CI** |
| --- | --- | --- | --- | --- | --- | --- | --- | --- | --- |
| Externally validated PPS original model | gender | Male | 769 | 575 | 194 | Balanced Accuracy | 0.69235491 | 0.65858359 | 0.72503731 |
| Externally validated PPS original model | gender | Male | 769 | 575 | 194 | Sensitivity | 0.54401652 | 0.5026087 | 0.58434783 |
| Externally validated PPS original model | gender | Male | 769 | 575 | 194 | Specificity | 0.8406933 | 0.78865979 | 0.89175258 |
| Externally validated PPS original model | gender | Male | 769 | 575 | 194 | PPV | 0.91016528 | 0.88169463 | 0.93636032 |
| Externally validated PPS original model | gender | Male | 769 | 575 | 194 | NPV | 0.38371165 | 0.35784562 | 0.41044453 |
| Externally validated PPS original model | gender | Male | 769 | 575 | 194 | Brier Score | 0.18424212 | 0.17554295 | 0.19210364 |
| Externally validated PPS original model | gender | Male | 769 | 575 | 194 | Calibration Intercept | -1.04680481 | -1.12444675 | -0.96862431 |
| Externally validated PPS original model | gender | Male | 769 | 575 | 194 | Calibration Slope | 0.8976523 | 0.72936785 | 1.1004707 |
| Externally validated PPS original model | gender | Male | 769 | 575 | 194 | ICI | 0.27630285 | 0.26794567 | 0.28442495 |
| Externally validated PPS original model | gender | Male | 769 | 575 | 194 | Harrell's C | 0.75618626 | 0.72020957 | 0.79146055 |
| Externally validated PPS original model | gender | Female | 1223 | 906 | 317 | Balanced Accuracy | 0.70414908 | 0.67736297 | 0.72948276 |
| Externally validated PPS original model | gender | Female | 1223 | 906 | 317 | Sensitivity | 0.54660099 | 0.51434879 | 0.57836645 |
| Externally validated PPS original model | gender | Female | 1223 | 906 | 317 | Specificity | 0.86169716 | 0.82334385 | 0.89905363 |
| Externally validated PPS original model | gender | Female | 1223 | 906 | 317 | PPV | 0.918714 | 0.89648959 | 0.93977825 |
| Externally validated PPS original model | gender | Female | 1223 | 906 | 317 | NPV | 0.39953729 | 0.37883459 | 0.42050351 |
| Externally validated PPS original model | gender | Female | 1223 | 906 | 317 | Brier Score | 0.17572118 | 0.16811083 | 0.18318936 |
| Externally validated PPS original model | gender | Female | 1223 | 906 | 317 | Calibration Intercept | -1.00578612 | -1.07531152 | -0.93891633 |
| Externally validated PPS original model | gender | Female | 1223 | 906 | 317 | Calibration Slope | 0.9528013 | 0.8154622 | 1.11091525 |
| Externally validated PPS original model | gender | Female | 1223 | 906 | 317 | ICI | 0.27174447 | 0.26401364 | 0.27919708 |
| Externally validated PPS original model | gender | Female | 1223 | 906 | 317 | Harrell's C | 0.77964185 | 0.75128725 | 0.8081417 |
| Externally validated PPS original model | ethnicity | White | 1234 | 960 | 274 | Balanced Accuracy | 0.73307576 | 0.70535893 | 0.7618855 |
| Externally validated PPS original model | ethnicity | White | 1234 | 960 | 274 | Sensitivity | 0.66669531 | 0.63645833 | 0.69580708 |
| Externally validated PPS original model | ethnicity | White | 1234 | 960 | 274 | Specificity | 0.7994562 | 0.75182482 | 0.85027298 |
| Externally validated PPS original model | ethnicity | White | 1234 | 960 | 274 | PPV | 0.92099224 | 0.90409075 | 0.93900709 |
| Externally validated PPS original model | ethnicity | White | 1234 | 960 | 274 | NPV | 0.40659029 | 0.38204877 | 0.43258296 |
| Externally validated PPS original model | ethnicity | White | 1234 | 960 | 274 | Brier Score | 0.16217818 | 0.15593362 | 0.16836998 |
| Externally validated PPS original model | ethnicity | White | 1234 | 960 | 274 | Calibration Intercept | -1.12795678 | -1.19318726 | -1.06017455 |
| Externally validated PPS original model | ethnicity | White | 1234 | 960 | 274 | Calibration Slope | 1.03146686 | 0.88775487 | 1.18629484 |
| Externally validated PPS original model | ethnicity | White | 1234 | 960 | 274 | ICI | 0.23945522 | 0.23346642 | 0.2453921 |
| Externally validated PPS original model | ethnicity | White | 1234 | 960 | 274 | Harrell's C | 0.79768725 | 0.77000589 | 0.82449762 |
| Externally validated PPS original model | ethnicity | Black | 169 | 137 | 32 | Balanced Accuracy | 0.65936736 | 0.5706235 | 0.74041971 |
| Externally validated PPS original model | ethnicity | Black | 169 | 137 | 32 | Sensitivity | 0.56832847 | 0.48905109 | 0.64963504 |
| Externally validated PPS original model | ethnicity | Black | 169 | 137 | 32 | Specificity | 0.75040625 | 0.59375 | 0.875 |
| Externally validated PPS original model | ethnicity | Black | 169 | 137 | 32 | PPV | 0.90730199 | 0.85334153 | 0.95555556 |
| Externally validated PPS original model | ethnicity | Black | 169 | 137 | 32 | NPV | 0.28956576 | 0.23333333 | 0.34848056 |
| Externally validated PPS original model | ethnicity | Black | 169 | 137 | 32 | Brier Score | 0.13815621 | 0.12146464 | 0.15282472 |
| Externally validated PPS original model | ethnicity | Black | 169 | 137 | 32 | Calibration Intercept | -0.78214898 | -0.94891937 | -0.62765324 |
| Externally validated PPS original model | ethnicity | Black | 169 | 137 | 32 | Calibration Slope | 0.9956644 | 0.60598131 | 1.47127236 |
| Externally validated PPS original model | ethnicity | Black | 169 | 137 | 32 | ICI | 0.22126368 | 0.20522177 | 0.2359565 |
| Externally validated PPS original model | ethnicity | Black | 169 | 137 | 32 | Harrell's C | 0.74286759 | 0.64338522 | 0.82880931 |
| Externally validated PPS original model | ethnicity | Other | 758 | 545 | 213 | Balanced Accuracy | 0.65384599 | 0.6242064 | 0.68008787 |
| Externally validated PPS original model | ethnicity | Other | 758 | 545 | 213 | Sensitivity | 0.38242202 | 0.3412844 | 0.42201835 |
| Externally validated PPS original model | ethnicity | Other | 758 | 545 | 213 | Specificity | 0.92526995 | 0.88732394 | 0.95774648 |
| Externally validated PPS original model | ethnicity | Other | 758 | 545 | 213 | PPV | 0.92913943 | 0.89427313 | 0.960348 |
| Externally validated PPS original model | ethnicity | Other | 758 | 545 | 213 | NPV | 0.36943134 | 0.35090996 | 0.3875969 |
| Externally validated PPS original model | ethnicity | Other | 758 | 545 | 213 | Brier Score | 0.18586549 | 0.17601351 | 0.1958367 |
| Externally validated PPS original model | ethnicity | Other | 758 | 545 | 213 | Calibration Intercept | -0.79353606 | -0.87082351 | -0.71477312 |
| Externally validated PPS original model | ethnicity | Other | 758 | 545 | 213 | Calibration Slope | 0.91814797 | 0.73244586 | 1.12514611 |
| Externally validated PPS original model | ethnicity | Other | 758 | 545 | 213 | ICI | 0.30239404 | 0.29194783 | 0.3129202 |
| Externally validated PPS original model | ethnicity | Other | 758 | 545 | 213 | Harrell's C | 0.76372732 | 0.726441 | 0.79836294 |
| Recalibrated PPS original model | gender | Male | 769 | 575 | 194 | Balanced Accuracy | 0.6972456 | 0.66161893 | 0.73153136 |
| Recalibrated PPS original model | gender | Male | 769 | 575 | 194 | Sensitivity | 0.61019739 | 0.57043478 | 0.64869565 |
| Recalibrated PPS original model | gender | Male | 769 | 575 | 194 | Specificity | 0.78429381 | 0.72680412 | 0.84523091 |
| Recalibrated PPS original model | gender | Male | 769 | 575 | 194 | PPV | 0.89353899 | 0.86732318 | 0.92030607 |
| Recalibrated PPS original model | gender | Male | 769 | 575 | 194 | NPV | 0.40459632 | 0.375 | 0.43444677 |
| Recalibrated PPS original model | gender | Male | 769 | 575 | 194 | Brier Score | 0.16302784 | 0.15351813 | 0.17227584 |
| Recalibrated PPS original model | gender | Male | 769 | 575 | 194 | Calibration Intercept | -0.0754346 | -0.14869254 | -0.00230522 |
| Recalibrated PPS original model | gender | Male | 769 | 575 | 194 | Calibration Slope | 0.95470452 | 0.77572438 | 1.17041347 |
| Recalibrated PPS original model | gender | Male | 769 | 575 | 194 | ICI | 0.31563953 | 0.30447147 | 0.32655028 |
| Recalibrated PPS original model | gender | Male | 769 | 575 | 194 | Harrell's C | 0.75618626 | 0.72020957 | 0.79146055 |
| Recalibrated PPS original model | gender | Female | 1223 | 906 | 317 | Balanced Accuracy | 0.71490356 | 0.6852463 | 0.74098497 |
| Recalibrated PPS original model | gender | Female | 1223 | 906 | 317 | Sensitivity | 0.60595695 | 0.57174393 | 0.63686534 |
| Recalibrated PPS original model | gender | Female | 1223 | 906 | 317 | Specificity | 0.82385016 | 0.77917981 | 0.86435331 |
| Recalibrated PPS original model | gender | Female | 1223 | 906 | 317 | PPV | 0.90772558 | 0.88603733 | 0.92833561 |
| Recalibrated PPS original model | gender | Female | 1223 | 906 | 317 | NPV | 0.42265788 | 0.39720282 | 0.44645353 |
| Recalibrated PPS original model | gender | Female | 1223 | 906 | 317 | Brier Score | 0.1552406 | 0.14688235 | 0.16362138 |
| Recalibrated PPS original model | gender | Female | 1223 | 906 | 317 | Calibration Intercept | -0.04042781 | -0.10561397 | 0.02217003 |
| Recalibrated PPS original model | gender | Female | 1223 | 906 | 317 | Calibration Slope | 1.01335863 | 0.86729065 | 1.18152184 |
| Recalibrated PPS original model | gender | Female | 1223 | 906 | 317 | ICI | 0.30743608 | 0.29716593 | 0.31709674 |
| Recalibrated PPS original model | gender | Female | 1223 | 906 | 317 | Harrell's C | 0.77964185 | 0.75128725 | 0.8081417 |
| Recalibrated PPS original model | ethnicity | White | 1234 | 960 | 274 | Balanced Accuracy | 0.73028087 | 0.70220508 | 0.75901754 |
| Recalibrated PPS original model | ethnicity | White | 1234 | 960 | 274 | Sensitivity | 0.69378802 | 0.66354167 | 0.721875 |
| Recalibrated PPS original model | ethnicity | White | 1234 | 960 | 274 | Specificity | 0.76677372 | 0.7189781 | 0.81751825 |
| Recalibrated PPS original model | ethnicity | White | 1234 | 960 | 274 | PPV | 0.91251523 | 0.8957526 | 0.92987377 |
| Recalibrated PPS original model | ethnicity | White | 1234 | 960 | 274 | NPV | 0.41702535 | 0.39047619 | 0.44535545 |
| Recalibrated PPS original model | ethnicity | White | 1234 | 960 | 274 | Brier Score | 0.14177594 | 0.13457888 | 0.14871705 |
| Recalibrated PPS original model | ethnicity | White | 1234 | 960 | 274 | Calibration Intercept | -0.14450074 | -0.2060147 | -0.08049414 |
| Recalibrated PPS original model | ethnicity | White | 1234 | 960 | 274 | Calibration Slope | 1.09702394 | 0.94417803 | 1.26169235 |
| Recalibrated PPS original model | ethnicity | White | 1234 | 960 | 274 | ICI | 0.27519455 | 0.26652348 | 0.28351173 |
| Recalibrated PPS original model | ethnicity | White | 1234 | 960 | 274 | Harrell's C | 0.79768725 | 0.77000589 | 0.82449762 |
| Recalibrated PPS original model | ethnicity | Black | 169 | 137 | 32 | Balanced Accuracy | 0.65647913 | 0.5608151 | 0.74566606 |
| Recalibrated PPS original model | ethnicity | Black | 169 | 137 | 32 | Sensitivity | 0.68592701 | 0.60583942 | 0.76623939 |
| Recalibrated PPS original model | ethnicity | Black | 169 | 137 | 32 | Specificity | 0.62703125 | 0.46875 | 0.78125 |
| Recalibrated PPS original model | ethnicity | Black | 169 | 137 | 32 | PPV | 0.88755876 | 0.84 | 0.93203883 |
| Recalibrated PPS original model | ethnicity | Black | 169 | 137 | 32 | NPV | 0.31918484 | 0.23882186 | 0.40350164 |
| Recalibrated PPS original model | ethnicity | Black | 169 | 137 | 32 | Brier Score | 0.13137833 | 0.11368391 | 0.14876087 |
| Recalibrated PPS original model | ethnicity | Black | 169 | 137 | 32 | Calibration Intercept | 0.19242185 | 0.03419806 | 0.33750063 |
| Recalibrated PPS original model | ethnicity | Black | 169 | 137 | 32 | Calibration Slope | 1.05894598 | 0.64449575 | 1.56478223 |
| Recalibrated PPS original model | ethnicity | Black | 169 | 137 | 32 | ICI | 0.27561988 | 0.25330489 | 0.29756603 |
| Recalibrated PPS original model | ethnicity | Black | 169 | 137 | 32 | Harrell's C | 0.74286759 | 0.64338522 | 0.82880931 |
| Recalibrated PPS original model | ethnicity | Other | 758 | 545 | 213 | Balanced Accuracy | 0.68242298 | 0.65097829 | 0.71381681 |
| Recalibrated PPS original model | ethnicity | Other | 758 | 545 | 213 | Sensitivity | 0.4864422 | 0.4440367 | 0.52844037 |
| Recalibrated PPS original model | ethnicity | Other | 758 | 545 | 213 | Specificity | 0.87840376 | 0.83098592 | 0.92018779 |
| Recalibrated PPS original model | ethnicity | Other | 758 | 545 | 213 | PPV | 0.9110958 | 0.8797564 | 0.941744 |
| Recalibrated PPS original model | ethnicity | Other | 758 | 545 | 213 | NPV | 0.40083611 | 0.37755209 | 0.42471377 |
| Recalibrated PPS original model | ethnicity | Other | 758 | 545 | 213 | Brier Score | 0.16996154 | 0.15916053 | 0.18111963 |
| Recalibrated PPS original model | ethnicity | Other | 758 | 545 | 213 | Calibration Intercept | 0.15526866 | 0.08231099 | 0.22973496 |
| Recalibrated PPS original model | ethnicity | Other | 758 | 545 | 213 | Calibration Slope | 0.97650283 | 0.77899802 | 1.19665718 |
| Recalibrated PPS original model | ethnicity | Other | 758 | 545 | 213 | ICI | 0.34471672 | 0.3320364 | 0.35790985 |
| Recalibrated PPS original model | ethnicity | Other | 758 | 545 | 213 | Harrell's C | 0.76372732 | 0.726441 | 0.79836294 |

**eTable 8**. Per-fold percentage of missing data within the refined PPS model.

| **Outer Repeat** | **Fold** | **Predictor** | **Missingness (%)** |
| --- | --- | --- | --- |
| 1 | 1 | paternal age | 46.27 |
| 1 | 1 | childhood social withdrawal | 49.16 |
| 1 | 2 | paternal age | 46.64 |
| 1 | 2 | childhood social withdrawal | 49.65 |
| 1 | 3 | paternal age | 46.39 |
| 1 | 3 | childhood social withdrawal | 49.97 |
| 1 | 4 | paternal age | 47.28 |
| 1 | 4 | childhood social withdrawal | 50.46 |
| 1 | 5 | paternal age | 46.65 |
| 1 | 5 | childhood social withdrawal | 50.64 |
| 2 | 1 | paternal age | 46.13 |
| 2 | 1 | childhood social withdrawal | 49.48 |
| 2 | 2 | paternal age | 47.08 |
| 2 | 2 | childhood social withdrawal | 49.91 |
| 2 | 3 | paternal age | 46.47 |
| 2 | 3 | childhood social withdrawal | 49.88 |
| 2 | 4 | paternal age | 46.35 |
| 2 | 4 | childhood social withdrawal | 50.06 |
| 2 | 5 | paternal age | 47.19 |
| 2 | 5 | childhood social withdrawal | 50.55 |
| 3 | 1 | paternal age | 47.05 |
| 3 | 1 | childhood social withdrawal | 50.29 |
| 3 | 2 | paternal age | 46.7 |
| 3 | 2 | childhood social withdrawal | 50.23 |
| 3 | 3 | paternal age | 46.91 |
| 3 | 3 | childhood social withdrawal | 49.8 |
| 3 | 4 | paternal age | 45.98 |
| 3 | 4 | childhood social withdrawal | 50.03 |
| 3 | 5 | paternal age | 46.59 |
| 3 | 5 | childhood social withdrawal | 49.54 |
| 4 | 1 | paternal age | 47.43 |
| 4 | 1 | childhood social withdrawal | 50.09 |
| 4 | 2 | paternal age | 46.1 |
| 4 | 2 | childhood social withdrawal | 49.74 |
| 4 | 3 | paternal age | 46.39 |
| 4 | 3 | childhood social withdrawal | 49.91 |
| 4 | 4 | paternal age | 47.34 |
| 4 | 4 | childhood social withdrawal | 50.23 |
| 4 | 5 | paternal age | 45.98 |
| 4 | 5 | childhood social withdrawal | 49.91 |
| 5 | 1 | paternal age | 46.62 |
| 5 | 1 | childhood social withdrawal | 49.8 |
| 5 | 2 | paternal age | 46.24 |
| 5 | 2 | childhood social withdrawal | 50.35 |
| 5 | 3 | paternal age | 46.64 |
| 5 | 3 | childhood social withdrawal | 49.71 |
| 5 | 4 | paternal age | 46.62 |
| 5 | 4 | childhood social withdrawal | 49.62 |
| 5 | 5 | paternal age | 47.11 |
| 5 | 5 | childhood social withdrawal | 50.41 |
| 6 | 1 | paternal age | 46.01 |
| 6 | 1 | childhood social withdrawal | 50.35 |
| 6 | 2 | paternal age | 47.02 |
| 6 | 2 | childhood social withdrawal | 49.62 |
| 6 | 3 | paternal age | 47.25 |
| 6 | 3 | childhood social withdrawal | 49.45 |
| 6 | 4 | paternal age | 46.99 |
| 6 | 4 | childhood social withdrawal | 50.98 |
| 6 | 5 | paternal age | 45.95 |
| 6 | 5 | childhood social withdrawal | 49.48 |
| 7 | 1 | paternal age | 46.62 |
| 7 | 1 | childhood social withdrawal | 49.97 |
| 7 | 2 | paternal age | 46.85 |
| 7 | 2 | childhood social withdrawal | 49.86 |
| 7 | 3 | paternal age | 46.62 |
| 7 | 3 | childhood social withdrawal | 49.86 |
| 7 | 4 | paternal age | 46.76 |
| 7 | 4 | childhood social withdrawal | 50.23 |
| 7 | 5 | paternal age | 46.39 |
| 7 | 5 | childhood social withdrawal | 49.97 |
| 8 | 1 | paternal age | 46.42 |
| 8 | 1 | childhood social withdrawal | 49.71 |
| 8 | 2 | paternal age | 47.43 |
| 8 | 2 | childhood social withdrawal | 49.97 |
| 8 | 3 | paternal age | 46.15 |
| 8 | 3 | childhood social withdrawal | 49.74 |
| 8 | 4 | paternal age | 46.7 |
| 8 | 4 | childhood social withdrawal | 50.29 |
| 8 | 5 | paternal age | 46.53 |
| 8 | 5 | childhood social withdrawal | 50.17 |
| 9 | 1 | paternal age | 46.39 |
| 9 | 1 | childhood social withdrawal | 50.09 |
| 9 | 2 | paternal age | 46.18 |
| 9 | 2 | childhood social withdrawal | 49.77 |
| 9 | 3 | paternal age | 47.45 |
| 9 | 3 | childhood social withdrawal | 49.54 |
| 9 | 4 | paternal age | 46.88 |
| 9 | 4 | childhood social withdrawal | 50.58 |
| 9 | 5 | paternal age | 46.33 |
| 9 | 5 | childhood social withdrawal | 49.91 |
| 10 | 1 | paternal age | 47.05 |
| 10 | 1 | childhood social withdrawal | 49.83 |
| 10 | 2 | paternal age | 46.39 |
| 10 | 2 | childhood social withdrawal | 50.38 |
| 10 | 3 | paternal age | 46.76 |
| 10 | 3 | childhood social withdrawal | 50.23 |
| 10 | 4 | paternal age | 46.44 |
| 10 | 4 | childhood social withdrawal | 48.93 |
| 10 | 5 | paternal age | 46.59 |
| 10 | 5 | childhood social withdrawal | 50.52 |

**eTable 9.** Per-fold classification indices from the refined PPS model using RN-CV^a^.

| **C_test** | **Fold** | **OuterRepeat** | **n_train** | **events_train** | **n_test** | **events_test** | **balanced_accuracy** | **sensitivity** | **specificity** | **ppv** | **npv** | **precision** | **recall** | **f1** |
| --- | --- | --- | --- | --- | --- | --- | --- | --- | --- | --- | --- | --- | --- | --- |
| 0.913 | 1 | 1 | 1729 | 1314 | 432 | 328 | 0.83184803 | 0.807927 | 0.855769 | 0.9 | 0.6 | 0.94643 | 0.81 | 1 |
| 0.845 | 2 | 1 | 1728 | 1313 | 433 | 329 | 0.740296937 | 0.826748 | 0.653846 | 0.9 | 0.5 | 0.88312 | 0.83 | 1 |
| 0.893 | 3 | 1 | 1729 | 1314 | 432 | 328 | 0.805933396 | 0.881098 | 0.730769 | 0.9 | 0.7 | 0.91167 | 0.88 | 1 |
| 0.864 | 4 | 1 | 1728 | 1313 | 433 | 329 | 0.792655483 | 0.787234 | 0.798077 | 0.9 | 0.5 | 0.925 | 0.79 | 1 |
| 0.857 | 5 | 1 | 1730 | 1314 | 431 | 328 | 0.774419844 | 0.810976 | 0.737864 | 0.9 | 0.6 | 0.90785 | 0.81 | 1 |
| 0.864 | 1 | 2 | 1730 | 1314 | 431 | 328 | 0.79993488 | 0.823171 | 0.776699 | 0.9 | 0.6 | 0.9215 | 0.82 | 1 |
| 0.868 | 2 | 2 | 1729 | 1314 | 432 | 328 | 0.754690432 | 0.817073 | 0.692308 | 0.9 | 0.5 | 0.89333 | 0.82 | 1 |
| 0.861 | 3 | 2 | 1728 | 1313 | 433 | 329 | 0.753419453 | 0.756839 | 0.75 | 0.9 | 0.5 | 0.90545 | 0.76 | 1 |
| 0.902 | 4 | 2 | 1728 | 1313 | 433 | 329 | 0.82708382 | 0.817629 | 0.836538 | 0.9 | 0.6 | 0.94056 | 0.82 | 1 |
| 0.876 | 5 | 2 | 1729 | 1314 | 432 | 328 | 0.797725141 | 0.82622 | 0.769231 | 0.9 | 0.6 | 0.91864 | 0.83 | 1 |
| 0.911 | 1 | 3 | 1728 | 1313 | 433 | 329 | 0.806274842 | 0.699088 | 0.913462 | 1 | 0.5 | 0.96234 | 0.7 | 1 |
| 0.863 | 2 | 3 | 1728 | 1313 | 433 | 329 | 0.758007949 | 0.823708 | 0.692308 | 0.9 | 0.6 | 0.89439 | 0.82 | 1 |
| 0.896 | 3 | 3 | 1729 | 1314 | 432 | 328 | 0.791510319 | 0.881098 | 0.701923 | 0.9 | 0.7 | 0.90313 | 0.88 | 1 |
| 0.872 | 4 | 3 | 1729 | 1314 | 432 | 328 | 0.779666979 | 0.780488 | 0.778846 | 0.9 | 0.5 | 0.91756 | 0.78 | 1 |
| 0.834 | 5 | 3 | 1730 | 1314 | 431 | 328 | 0.76098153 | 0.77439 | 0.747573 | 0.9 | 0.5 | 0.90714 | 0.77 | 1 |
| 0.901 | 1 | 4 | 1729 | 1314 | 432 | 328 | 0.820356473 | 0.756098 | 0.884615 | 1 | 0.5 | 0.95385 | 0.76 | 1 |
| 0.872 | 2 | 4 | 1729 | 1314 | 432 | 328 | 0.761139775 | 0.878049 | 0.644231 | 0.9 | 0.6 | 0.88615 | 0.88 | 1 |
| 0.86 | 3 | 4 | 1729 | 1313 | 432 | 329 | 0.769601912 | 0.869301 | 0.669903 | 0.9 | 0.6 | 0.89375 | 0.87 | 1 |
| 0.901 | 4 | 4 | 1728 | 1313 | 433 | 329 | 0.829100421 | 0.802432 | 0.855769 | 0.9 | 0.6 | 0.94624 | 0.8 | 1 |
| 0.845 | 5 | 4 | 1729 | 1314 | 432 | 328 | 0.749179174 | 0.844512 | 0.653846 | 0.9 | 0.6 | 0.88498 | 0.84 | 1 |
| 0.854 | 1 | 5 | 1729 | 1314 | 432 | 328 | 0.774038462 | 0.875 | 0.673077 | 0.9 | 0.6 | 0.89408 | 0.88 | 1 |
| 0.844 | 2 | 5 | 1730 | 1314 | 431 | 328 | 0.740158063 | 0.820122 | 0.660194 | 0.9 | 0.5 | 0.88487 | 0.82 | 1 |
| 0.889 | 3 | 5 | 1728 | 1313 | 433 | 329 | 0.800283493 | 0.860182 | 0.740385 | 0.9 | 0.6 | 0.9129 | 0.86 | 1 |
| 0.886 | 4 | 5 | 1729 | 1314 | 432 | 328 | 0.779666979 | 0.780488 | 0.778846 | 0.9 | 0.5 | 0.91756 | 0.78 | 1 |
| 0.889 | 5 | 5 | 1728 | 1313 | 433 | 329 | 0.795139701 | 0.696049 | 0.894231 | 1 | 0.5 | 0.95417 | 0.7 | 1 |
| 0.883 | 1 | 6 | 1728 | 1313 | 433 | 329 | 0.78127192 | 0.793313 | 0.769231 | 0.9 | 0.5 | 0.91579 | 0.79 | 1 |
| 0.843 | 2 | 6 | 1729 | 1314 | 432 | 328 | 0.751641651 | 0.810976 | 0.692308 | 0.9 | 0.5 | 0.89262 | 0.81 | 1 |
| 0.898 | 3 | 6 | 1729 | 1314 | 432 | 328 | 0.80956848 | 0.801829 | 0.817308 | 0.9 | 0.6 | 0.93262 | 0.8 | 1 |
| 0.88 | 4 | 6 | 1730 | 1314 | 431 | 328 | 0.78745856 | 0.807927 | 0.76699 | 0.9 | 0.6 | 0.91696 | 0.81 | 1 |
| 0.87 | 5 | 6 | 1728 | 1313 | 433 | 329 | 0.775748188 | 0.878419 | 0.673077 | 0.9 | 0.6 | 0.89474 | 0.88 | 1 |
| 0.864 | 1 | 7 | 1729 | 1313 | 432 | 329 | 0.772641426 | 0.87538 | 0.669903 | 0.9 | 0.6 | 0.89441 | 0.88 | 1 |
| 0.869 | 2 | 7 | 1729 | 1314 | 432 | 328 | 0.768527205 | 0.777439 | 0.759615 | 0.9 | 0.5 | 0.91071 | 0.78 | 1 |
| 0.89 | 3 | 7 | 1729 | 1314 | 432 | 328 | 0.796904315 | 0.795732 | 0.798077 | 0.9 | 0.6 | 0.92553 | 0.8 | 1 |
| 0.867 | 4 | 7 | 1728 | 1313 | 433 | 329 | 0.778758476 | 0.826748 | 0.730769 | 0.9 | 0.6 | 0.90667 | 0.83 | 1 |
| 0.873 | 5 | 7 | 1729 | 1314 | 432 | 328 | 0.787640713 | 0.844512 | 0.730769 | 0.9 | 0.6 | 0.9082 | 0.84 | 1 |
| 0.875 | 1 | 8 | 1730 | 1314 | 431 | 328 | 0.762905517 | 0.817073 | 0.708738 | 0.9 | 0.5 | 0.89933 | 0.82 | 1 |
| 0.881 | 2 | 8 | 1729 | 1314 | 432 | 328 | 0.784122889 | 0.856707 | 0.711538 | 0.9 | 0.6 | 0.90354 | 0.86 | 1 |
| 0.847 | 3 | 8 | 1729 | 1314 | 432 | 328 | 0.743316135 | 0.823171 | 0.663462 | 0.9 | 0.5 | 0.88525 | 0.82 | 1 |
| 0.912 | 4 | 8 | 1728 | 1313 | 433 | 329 | 0.806830138 | 0.796353 | 0.817308 | 0.9 | 0.6 | 0.93238 | 0.8 | 1 |
| 0.85 | 5 | 8 | 1728 | 1313 | 433 | 329 | 0.775967384 | 0.81155 | 0.740385 | 0.9 | 0.6 | 0.90816 | 0.81 | 1 |
| 0.864 | 1 | 9 | 1729 | 1314 | 432 | 328 | 0.789868668 | 0.820122 | 0.759615 | 0.9 | 0.6 | 0.91497 | 0.82 | 1 |
| 0.871 | 2 | 9 | 1730 | 1314 | 431 | 328 | 0.773058252 | 0.75 | 0.796117 | 0.9 | 0.5 | 0.92135 | 0.75 | 1 |
| 0.917 | 3 | 9 | 1728 | 1313 | 433 | 329 | 0.794613631 | 0.656535 | 0.932692 | 1 | 0.5 | 0.96861 | 0.66 | 1 |
| 0.849 | 4 | 9 | 1728 | 1313 | 433 | 329 | 0.750131517 | 0.759878 | 0.740385 | 0.9 | 0.5 | 0.90253 | 0.76 | 1 |
| 0.862 | 5 | 9 | 1729 | 1314 | 432 | 328 | 0.767589118 | 0.814024 | 0.721154 | 0.9 | 0.6 | 0.90203 | 0.81 | 1 |
| 0.901 | 1 | 10 | 1730 | 1314 | 431 | 328 | 0.812574 | 0.780488 | 0.84466 | 0.9 | 0.5 | 0.94118 | 0.78 | 1 |
| 0.894 | 2 | 10 | 1729 | 1314 | 432 | 328 | 0.803705441 | 0.780488 | 0.826923 | 0.9 | 0.5 | 0.93431 | 0.78 | 1 |
| 0.843 | 3 | 10 | 1728 | 1313 | 433 | 329 | 0.743088029 | 0.841945 | 0.644231 | 0.9 | 0.6 | 0.88217 | 0.84 | 1 |
| 0.857 | 4 | 10 | 1729 | 1314 | 432 | 328 | 0.765830206 | 0.820122 | 0.711538 | 0.9 | 0.6 | 0.89967 | 0.82 | 1 |
| 0.893 | 5 | 10 | 1728 | 1313 | 433 | 329 | 0.820785597 | 0.87234 | 0.769231 | 0.9 | 0.7 | 0.92283 | 0.87 | 1 |

^a^RN-CV = repeated nested cross validation

**eTable 10.** Per-fold calibration indices from the refined PPS model using RN-CV^a^.

| **fold** | **OuterRepeat** | **intercept** | **slope** | **brier** | **ICI** |
| --- | --- | --- | --- | --- | --- |
| 1 | 1 | 0.07102 | 1.35 | 0.1 | 0 |
| 2 | 1 | -0.14134 | 0.85 | 0.13 | 0 |
| 3 | 1 | -0.02791 | 1.18 | 0.11 | 0 |
| 4 | 1 | 0.15888 | 1.02 | 0.12 | 0 |
| 5 | 1 | -0.02631 | 0.8 | 0.12 | 0 |
| 1 | 2 | 0.19707 | 1.01 | 0.12 | 0 |
| 2 | 2 | -0.1156 | 0.94 | 0.12 | 0 |
| 3 | 2 | -0.01247 | 0.94 | 0.12 | 0 |
| 4 | 2 | -0.01504 | 1.23 | 0.11 | 0 |
| 5 | 2 | -0.08267 | 0.98 | 0.11 | 0 |
| 1 | 3 | 0.03642 | 1.31 | 0.1 | 0 |
| 2 | 3 | -0.072 | 0.87 | 0.12 | 0 |
| 3 | 3 | -0.1043 | 1.34 | 0.11 | 0 |
| 4 | 3 | 0.0812 | 0.94 | 0.12 | 0 |
| 5 | 3 | 0.1365 | 0.8 | 0.13 | 0 |
| 1 | 4 | 0.08306 | 1.3 | 0.1 | 0 |
| 2 | 4 | -0.28445 | 0.96 | 0.12 | 0 |
| 3 | 4 | -0.08435 | 0.87 | 0.12 | 0 |
| 4 | 4 | 0.26462 | 1.24 | 0.1 | 0 |
| 5 | 4 | -0.0156 | 0.87 | 0.13 | 0 |
| 1 | 5 | -0.08387 | 0.87 | 0.12 | 0 |
| 2 | 5 | 0.02721 | 0.88 | 0.12 | 0 |
| 3 | 5 | -0.12665 | 1.18 | 0.11 | 0 |
| 4 | 5 | -0.05625 | 1.03 | 0.11 | 0 |
| 5 | 5 | 0.19996 | 1.09 | 0.11 | 0 |
| 1 | 6 | 0.10415 | 1.14 | 0.11 | 0 |
| 2 | 6 | 0.02586 | 0.96 | 0.12 | 0 |
| 3 | 6 | -0.00239 | 1.22 | 0.1 | 0 |
| 4 | 6 | 0.04603 | 0.93 | 0.12 | 0 |
| 5 | 6 | -0.23254 | 0.88 | 0.12 | 0 |
| 1 | 7 | -0.12013 | 0.97 | 0.12 | 0 |
| 2 | 7 | 0.09377 | 1.09 | 0.11 | 0 |
| 3 | 7 | -0.08535 | 1.19 | 0.1 | 0 |
| 4 | 7 | 0.08087 | 0.9 | 0.12 | 0 |
| 5 | 7 | -0.00611 | 0.96 | 0.12 | 0 |
| 1 | 8 | -0.06789 | 1.18 | 0.11 | 0 |
| 2 | 8 | -0.08321 | 0.91 | 0.12 | 0 |
| 3 | 8 | 0.1196 | 0.82 | 0.13 | 0 |
| 4 | 8 | 0.04098 | 1.42 | 0.1 | 0 |
| 5 | 8 | 0.06622 | 0.87 | 0.12 | 0 |
| 1 | 9 | 0.08275 | 0.92 | 0.12 | 0 |
| 2 | 9 | -0.01134 | 1.05 | 0.11 | 0 |
| 3 | 9 | 0.06917 | 1.42 | 0.09 | 0 |
| 4 | 9 | -0.10578 | 0.83 | 0.13 | 0 |
| 5 | 9 | -0.01651 | 1.01 | 0.12 | 0 |
| 1 | 10 | 0.10924 | 1.36 | 0.1 | 0 |
| 2 | 10 | 0.10718 | 1.11 | 0.11 | 0 |
| 3 | 10 | -0.21277 | 0.78 | 0.13 | 0 |
| 4 | 10 | -0.1265 | 1.04 | 0.12 | 0 |
| 5 | 10 | 0.10173 | 1.06 | 0.11 | 0 |

^a^RN-CV = repeated nested cross validation

**eTable 11.** Per-fold classification indices from the retrained PPS model using RN-CV^a^.

| **C_test** | **Fold** | **OuterRepeat** | **n_train** | **events_train** | **n_test** | **events_test** | **balanced_accuracy** | **sensitivity** | **specificity** | **ppv** | **npv** | **precision** | **recall** | **f1** |
| --- | --- | --- | --- | --- | --- | --- | --- | --- | --- | --- | --- | --- | --- | --- |
| 0.823 | 1 | 1 | 1729 | 1314 | 432 | 328 | 0.720919325 | 0.634146 | 0.807692 | 0.9 | 0.4 | 0.91228 | 0.63 | 1 |
| 0.789 | 2 | 1 | 1728 | 1313 | 433 | 329 | 0.703764321 | 0.638298 | 0.769231 | 0.9 | 0.4 | 0.89744 | 0.64 | 1 |
| 0.809 | 3 | 1 | 1729 | 1314 | 432 | 328 | 0.725140713 | 0.594512 | 0.855769 | 0.9 | 0.4 | 0.92857 | 0.59 | 1 |
| 0.792 | 4 | 1 | 1728 | 1313 | 433 | 329 | 0.71210837 | 0.62614 | 0.798077 | 0.9 | 0.4 | 0.90749 | 0.63 | 1 |
| 0.783 | 5 | 1 | 1730 | 1314 | 431 | 328 | 0.711520246 | 0.646341 | 0.776699 | 0.9 | 0.4 | 0.90213 | 0.65 | 1 |
| 0.772 | 1 | 2 | 1730 | 1314 | 431 | 328 | 0.703217499 | 0.60061 | 0.805825 | 0.9 | 0.4 | 0.90783 | 0.6 | 1 |
| 0.791 | 2 | 2 | 1729 | 1314 | 432 | 328 | 0.729362101 | 0.679878 | 0.778846 | 0.9 | 0.4 | 0.9065 | 0.68 | 1 |
| 0.801 | 3 | 2 | 1728 | 1313 | 433 | 329 | 0.734656301 | 0.680851 | 0.788462 | 0.9 | 0.4 | 0.91057 | 0.68 | 1 |
| 0.788 | 4 | 2 | 1728 | 1313 | 433 | 329 | 0.694894202 | 0.610942 | 0.778846 | 0.9 | 0.4 | 0.89732 | 0.61 | 1 |
| 0.828 | 5 | 2 | 1729 | 1314 | 432 | 328 | 0.756918386 | 0.667683 | 0.846154 | 0.9 | 0.4 | 0.93191 | 0.67 | 1 |
| 0.804 | 1 | 3 | 1728 | 1313 | 433 | 329 | 0.741977437 | 0.647416 | 0.836538 | 0.9 | 0.4 | 0.92609 | 0.65 | 1 |
| 0.805 | 2 | 3 | 1728 | 1313 | 433 | 329 | 0.72808043 | 0.68693 | 0.769231 | 0.9 | 0.4 | 0.904 | 0.69 | 1 |
| 0.832 | 3 | 3 | 1729 | 1314 | 432 | 328 | 0.74706848 | 0.676829 | 0.817308 | 0.9 | 0.4 | 0.92116 | 0.68 | 1 |
| 0.796 | 4 | 3 | 1729 | 1314 | 432 | 328 | 0.722443715 | 0.637195 | 0.807692 | 0.9 | 0.4 | 0.91266 | 0.64 | 1 |
| 0.75 | 5 | 3 | 1730 | 1314 | 431 | 328 | 0.660371774 | 0.573171 | 0.747573 | 0.9 | 0.4 | 0.8785 | 0.57 | 1 |
| 0.801 | 1 | 4 | 1729 | 1314 | 432 | 328 | 0.727603189 | 0.685976 | 0.769231 | 0.9 | 0.4 | 0.90361 | 0.69 | 1 |
| 0.791 | 2 | 4 | 1729 | 1314 | 432 | 328 | 0.720802064 | 0.70122 | 0.740385 | 0.9 | 0.4 | 0.89494 | 0.7 | 1 |
| 0.814 | 3 | 4 | 1729 | 1313 | 432 | 329 | 0.764231121 | 0.635258 | 0.893204 | 1 | 0.4 | 0.95 | 0.64 | 1 |
| 0.809 | 4 | 4 | 1728 | 1313 | 433 | 329 | 0.719429507 | 0.592705 | 0.846154 | 0.9 | 0.4 | 0.92417 | 0.59 | 1 |
| 0.793 | 5 | 4 | 1729 | 1314 | 432 | 328 | 0.711655722 | 0.682927 | 0.740385 | 0.9 | 0.4 | 0.89243 | 0.68 | 1 |
| 0.789 | 1 | 5 | 1729 | 1314 | 432 | 328 | 0.719277674 | 0.698171 | 0.740385 | 0.9 | 0.4 | 0.89453 | 0.7 | 1 |
| 0.769 | 2 | 5 | 1730 | 1314 | 431 | 328 | 0.703779896 | 0.582317 | 0.825243 | 0.9 | 0.4 | 0.91388 | 0.58 | 1 |
| 0.791 | 3 | 5 | 1728 | 1313 | 433 | 329 | 0.694148936 | 0.638298 | 0.75 | 0.9 | 0.4 | 0.88983 | 0.64 | 1 |
| 0.812 | 4 | 5 | 1729 | 1314 | 432 | 328 | 0.729596623 | 0.670732 | 0.788462 | 0.9 | 0.4 | 0.90909 | 0.67 | 1 |
| 0.819 | 5 | 5 | 1728 | 1313 | 433 | 329 | 0.770823591 | 0.647416 | 0.894231 | 1 | 0.4 | 0.95089 | 0.65 | 1 |
| 0.841 | 1 | 6 | 1728 | 1313 | 433 | 329 | 0.738660276 | 0.592705 | 0.884615 | 0.9 | 0.4 | 0.94203 | 0.59 | 1 |
| 0.761 | 2 | 6 | 1729 | 1314 | 432 | 328 | 0.685272045 | 0.64939 | 0.721154 | 0.9 | 0.4 | 0.88017 | 0.65 | 1 |
| 0.821 | 3 | 6 | 1729 | 1314 | 432 | 328 | 0.715173546 | 0.670732 | 0.759615 | 0.9 | 0.4 | 0.89796 | 0.67 | 1 |
| 0.817 | 4 | 6 | 1730 | 1314 | 431 | 328 | 0.756171561 | 0.667683 | 0.84466 | 0.9 | 0.4 | 0.93191 | 0.67 | 1 |
| 0.755 | 5 | 6 | 1728 | 1313 | 433 | 329 | 0.679229016 | 0.656535 | 0.701923 | 0.9 | 0.4 | 0.87449 | 0.66 | 1 |
| 0.783 | 1 | 7 | 1729 | 1313 | 432 | 329 | 0.698084811 | 0.629179 | 0.76699 | 0.9 | 0.4 | 0.8961 | 0.63 | 1 |
| 0.808 | 2 | 7 | 1729 | 1314 | 432 | 328 | 0.725257974 | 0.652439 | 0.798077 | 0.9 | 0.4 | 0.91064 | 0.65 | 1 |
| 0.795 | 3 | 7 | 1729 | 1314 | 432 | 328 | 0.709545028 | 0.640244 | 0.778846 | 0.9 | 0.4 | 0.90129 | 0.64 | 1 |
| 0.808 | 4 | 7 | 1728 | 1313 | 433 | 329 | 0.729322539 | 0.641337 | 0.817308 | 0.9 | 0.4 | 0.91739 | 0.64 | 1 |
| 0.798 | 5 | 7 | 1729 | 1314 | 432 | 328 | 0.74249531 | 0.667683 | 0.817308 | 0.9 | 0.4 | 0.92017 | 0.67 | 1 |
| 0.768 | 1 | 8 | 1730 | 1314 | 431 | 328 | 0.686286408 | 0.625 | 0.747573 | 0.9 | 0.4 | 0.88745 | 0.63 | 1 |
| 0.79 | 2 | 8 | 1729 | 1314 | 432 | 328 | 0.724554409 | 0.679878 | 0.769231 | 0.9 | 0.4 | 0.90283 | 0.68 | 1 |
| 0.805 | 3 | 8 | 1729 | 1314 | 432 | 328 | 0.735811445 | 0.615854 | 0.855769 | 0.9 | 0.4 | 0.93088 | 0.62 | 1 |
| 0.862 | 4 | 8 | 1728 | 1313 | 433 | 329 | 0.777180267 | 0.708207 | 0.846154 | 0.9 | 0.5 | 0.93574 | 0.71 | 1 |
| 0.761 | 5 | 8 | 1728 | 1313 | 433 | 329 | 0.69718845 | 0.644377 | 0.75 | 0.9 | 0.4 | 0.89076 | 0.64 | 1 |
| 0.775 | 1 | 9 | 1729 | 1314 | 432 | 328 | 0.710834897 | 0.652439 | 0.769231 | 0.9 | 0.4 | 0.89916 | 0.65 | 1 |
| 0.838 | 2 | 9 | 1730 | 1314 | 431 | 328 | 0.7655991 | 0.676829 | 0.854369 | 0.9 | 0.5 | 0.93671 | 0.68 | 1 |
| 0.834 | 3 | 9 | 1728 | 1313 | 433 | 329 | 0.746039864 | 0.674772 | 0.817308 | 0.9 | 0.4 | 0.92116 | 0.67 | 1 |
| 0.768 | 4 | 9 | 1728 | 1313 | 433 | 329 | 0.695171849 | 0.659574 | 0.730769 | 0.9 | 0.4 | 0.88571 | 0.66 | 1 |
| 0.769 | 5 | 9 | 1729 | 1314 | 432 | 328 | 0.683278612 | 0.664634 | 0.701923 | 0.9 | 0.4 | 0.8755 | 0.66 | 1 |
| 0.817 | 1 | 10 | 1730 | 1314 | 431 | 328 | 0.736635686 | 0.618902 | 0.854369 | 0.9 | 0.4 | 0.93119 | 0.62 | 1 |
| 0.82 | 2 | 10 | 1729 | 1314 | 432 | 328 | 0.737453096 | 0.676829 | 0.798077 | 0.9 | 0.4 | 0.91358 | 0.68 | 1 |
| 0.775 | 3 | 10 | 1728 | 1313 | 433 | 329 | 0.712882862 | 0.656535 | 0.769231 | 0.9 | 0.4 | 0.9 | 0.66 | 1 |
| 0.79 | 4 | 10 | 1729 | 1314 | 432 | 328 | 0.71869137 | 0.658537 | 0.778846 | 0.9 | 0.4 | 0.90377 | 0.66 | 1 |
| 0.799 | 5 | 10 | 1728 | 1313 | 433 | 329 | 0.729322539 | 0.641337 | 0.817308 | 0.9 | 0.4 | 0.91739 | 0.64 | 1 |

^a^RN-CV = repeated nested cross validation

**eTable 12.** Per-fold calibration indices from the retrained PPS model using RN-CV^a^.

| **fold** | **OuterRepeat** | **intercept** | **slope** | **brier** | **ICI** |
| --- | --- | --- | --- | --- | --- |
| 1 | 1 | 0.08889 | 1.38 | 0.14 | 0 |
| 2 | 1 | -0.14425 | 0.93 | 0.15 | 0 |
| 3 | 1 | -0.02512 | 1.38 | 0.14 | 0 |
| 4 | 1 | 0.09012 | 1.11 | 0.14 | 0 |
| 5 | 1 | -0.03608 | 1.04 | 0.15 | 0 |
| 1 | 2 | 0.08016 | 1.06 | 0.15 | 0 |
| 2 | 2 | -0.0553 | 0.99 | 0.15 | 0 |
| 3 | 2 | -0.08585 | 1.06 | 0.14 | 0 |
| 4 | 2 | 0.03021 | 1.24 | 0.15 | 0 |
| 5 | 2 | 0.02036 | 1.39 | 0.14 | 0 |
| 1 | 3 | 0.02404 | 1.27 | 0.14 | 0 |
| 2 | 3 | -0.09938 | 1.15 | 0.14 | 0 |
| 3 | 3 | -0.04009 | 1.79 | 0.14 | 0 |
| 4 | 3 | 0.01296 | 1 | 0.14 | 0 |
| 5 | 3 | 0.07053 | 0.82 | 0.15 | 0 |
| 1 | 4 | -0.00958 | 1.24 | 0.14 | 0 |
| 2 | 4 | -0.09081 | 1.17 | 0.15 | 0 |
| 3 | 4 | 0.04009 | 1.25 | 0.14 | 0 |
| 4 | 4 | 0.1211 | 1.41 | 0.14 | 0 |
| 5 | 4 | -0.08901 | 0.96 | 0.14 | 0 |
| 1 | 5 | -0.1033 | 1.03 | 0.15 | 0 |
| 2 | 5 | 0.12386 | 0.95 | 0.15 | 0 |
| 3 | 5 | -0.0013 | 1.15 | 0.14 | 0 |
| 4 | 5 | -0.08589 | 1.17 | 0.14 | 0 |
| 5 | 5 | 0.07606 | 1.17 | 0.14 | 0 |
| 1 | 6 | 0.11088 | 1.5 | 0.14 | 0 |
| 2 | 6 | 0.01137 | 1.04 | 0.15 | 0 |
| 3 | 6 | -0.01894 | 1.36 | 0.14 | 0 |
| 4 | 6 | 0.00423 | 1.13 | 0.14 | 0 |
| 5 | 6 | -0.13287 | 0.85 | 0.15 | 0 |
| 1 | 7 | 0.05829 | 1.08 | 0.15 | 0 |
| 2 | 7 | 0.09038 | 1.25 | 0.14 | 0 |
| 3 | 7 | -0.09469 | 0.93 | 0.14 | 0 |
| 4 | 7 | -0.02286 | 1.27 | 0.14 | 0 |
| 5 | 7 | -0.09303 | 1.06 | 0.15 | 0 |
| 1 | 8 | -0.04894 | 1.03 | 0.15 | 0 |
| 2 | 8 | -0.01237 | 0.95 | 0.15 | 0 |
| 3 | 8 | 0.1171 | 1.26 | 0.14 | 0 |
| 4 | 8 | -0.0426 | 1.85 | 0.13 | 0 |
| 5 | 8 | -0.00582 | 0.8 | 0.15 | 0 |
| 1 | 9 | -0.04048 | 1.14 | 0.15 | 0 |
| 2 | 9 | -0.00575 | 1.57 | 0.13 | 0 |
| 3 | 9 | 0.00542 | 1.48 | 0.13 | 0 |
| 4 | 9 | -0.08591 | 0.98 | 0.15 | 0 |
| 5 | 9 | 0.13297 | 0.92 | 0.15 | 0 |
| 1 | 10 | 0.02575 | 1.41 | 0.14 | 0 |
| 2 | 10 | 0.00232 | 1.41 | 0.14 | 0 |
| 3 | 10 | -0.03693 | 1.01 | 0.15 | 0 |
| 4 | 10 | -0.05328 | 1.05 | 0.14 | 0 |
| 5 | 10 | 0.02422 | 1.36 | 0.14 | 0 |

^a^RN-CV = repeated nested cross validation

**eTable 13.** Subgroup performance of the refined PPS model in AMP-SCZ by gender and ethnicity.

| **Model** | **Subgroup_variable** | **Subgroup** | **N** | **N_CHR** | **N_control** | **Metric** | **Estimate** | **Lower_95CI** | **Upper_95CI** |
| --- | --- | --- | --- | --- | --- | --- | --- | --- | --- |
| Refined PPS model | gender | Female | 1197 | 884 | 313 | C | 0.878713515 | 0.857484928 | 0.899306009 |
| Refined PPS model | gender | Female | 1197 | 884 | 313 | BalancedAccuracy | 0.763554783 | 0.734433988 | 0.792249954 |
| Refined PPS model | gender | Female | 1197 | 884 | 313 | Sensitivity | 0.926470588 | 0.909587352 | 0.942666265 |
| Refined PPS model | gender | Female | 1197 | 884 | 313 | Specificity | 0.600638978 | 0.544063358 | 0.654978744 |
| Refined PPS model | gender | Female | 1197 | 884 | 313 | PPV | 0.867584746 | 0.844827586 | 0.889364502 |
| Refined PPS model | gender | Female | 1197 | 884 | 313 | NPV | 0.743083004 | 0.688628571 | 0.79296875 |
| Refined PPS model | gender | Female | 1197 | 884 | 313 | Intercept | -0.015499697 | -0.1690107 | 0.14094425 |
| Refined PPS model | gender | Female | 1197 | 884 | 313 | Slope | 1.07781776 | 0.967471283 | 1.206403681 |
| Refined PPS model | gender | Female | 1197 | 884 | 313 | Brier | 0.117245985 | 0.106180657 | 0.129298678 |
| Refined PPS model | gender | Female | 1197 | 884 | 313 | ICI | 0.017559988 | 0.011120464 | 0.037407141 |
| Refined PPS model | gender | Male | 761 | 568 | 193 | C | 0.858206232 | 0.827316016 | 0.886737574 |
| Refined PPS model | gender | Male | 761 | 568 | 193 | BalancedAccuracy | 0.737489053 | 0.701463991 | 0.774583077 |
| Refined PPS model | gender | Male | 761 | 568 | 193 | Sensitivity | 0.910211268 | 0.887468512 | 0.932849685 |
| Refined PPS model | gender | Male | 761 | 568 | 193 | Specificity | 0.564766839 | 0.493007319 | 0.634527838 |
| Refined PPS model | gender | Male | 761 | 568 | 193 | PPV | 0.860232945 | 0.833607908 | 0.887960211 |
| Refined PPS model | gender | Male | 761 | 568 | 193 | NPV | 0.68125 | 0.608083977 | 0.753272727 |
| Refined PPS model | gender | Male | 761 | 568 | 193 | Intercept | 0.002253178 | -0.196361101 | 0.214526732 |
| Refined PPS model | gender | Male | 761 | 568 | 193 | Slope | 0.962022765 | 0.822766274 | 1.131122867 |
| Refined PPS model | gender | Male | 761 | 568 | 193 | Brier | 0.125062328 | 0.110473277 | 0.139719708 |
| Refined PPS model | gender | Male | 761 | 568 | 193 | ICI | 0.017152859 | 0.012689396 | 0.04205363 |
| Refined PPS model | gender | Non-binary | 140 | 132 | 8 | C | 0.836174242 | 0.744607955 | 0.917275051 |
| Refined PPS model | gender | Non-binary | 140 | 132 | 8 | BalancedAccuracy | 0.492424242 | 0.480916031 | 0.5 |
| Refined PPS model | gender | Non-binary | 140 | 132 | 8 | Sensitivity | 0.984848485 | 0.961832061 | 1 |
| Refined PPS model | gender | Non-binary | 140 | 132 | 8 | Specificity | 0 | 0 | 0 |
| Refined PPS model | gender | Non-binary | 140 | 132 | 8 | PPV | 0.942028986 | 0.898550725 | 0.97826087 |
| Refined PPS model | gender | Non-binary | 140 | 132 | 8 | NPV | 0 | 0 | 0 |
| Refined PPS model | gender | Non-binary | 140 | 132 | 8 | Intercept | 0.279292839 | -0.452661552 | 1.367685746 |
| Refined PPS model | gender | Non-binary | 140 | 132 | 8 | Slope | 0.777389534 | 0.465619286 | 1.37481759 |
| Refined PPS model | gender | Non-binary | 140 | 132 | 8 | Brier | 0.055872427 | 0.028721557 | 0.088811947 |
| Refined PPS model | gender | Non-binary | 140 | 132 | 8 | ICI | 0.030496442 | 0.025286687 | 0.078915474 |
| Refined PPS model | gender | Other | 29 | 29 | 0 | C | / | / | / |
| Refined PPS model | gender | Other | 29 | 29 | 0 | BalancedAccuracy | / | / | / |
| Refined PPS model | gender | Other | 29 | 29 | 0 | Sensitivity | / | / | / |
| Refined PPS model | gender | Other | 29 | 29 | 0 | Specificity | / | / | / |
| Refined PPS model | gender | Other | 29 | 29 | 0 | PPV | / | / | / |
| Refined PPS model | gender | Other | 29 | 29 | 0 | NPV | / | / | / |
| Refined PPS model | gender | Other | 29 | 29 | 0 | Intercept | / | / | / |
| Refined PPS model | gender | Other | 29 | 29 | 0 | Slope | / | / | / |
| Refined PPS model | gender | Other | 29 | 29 | 0 | Brier | / | / | / |
| Refined PPS model | gender | Other | 29 | 29 | 0 | ICI | / | / | / |
| Refined PPS model | ethnicity | Black | 169 | 137 | 32 | C | 0.820711679 | 0.739064039 | 0.894833994 |
| Refined PPS model | ethnicity | Black | 169 | 137 | 32 | BalancedAccuracy | 0.661952555 | 0.574871572 | 0.752789577 |
| Refined PPS model | ethnicity | Black | 169 | 137 | 32 | Sensitivity | 0.948905109 | 0.909722222 | 0.979865772 |
| Refined PPS model | ethnicity | Black | 169 | 137 | 32 | Specificity | 0.375 | 0.210471491 | 0.551746824 |
| Refined PPS model | ethnicity | Black | 169 | 137 | 32 | PPV | 0.866666667 | 0.80952381 | 0.918918919 |
| Refined PPS model | ethnicity | Black | 169 | 137 | 32 | NPV | 0.631578947 | 0.4 | 0.851984127 |
| Refined PPS model | ethnicity | Black | 169 | 137 | 32 | Intercept | 0.000179544 | -0.450264277 | 0.501661593 |
| Refined PPS model | ethnicity | Black | 169 | 137 | 32 | Slope | 0.86505775 | 0.590469148 | 1.273933881 |
| Refined PPS model | ethnicity | Black | 169 | 137 | 32 | Brier | 0.116902145 | 0.084982353 | 0.149510213 |
| Refined PPS model | ethnicity | Black | 169 | 137 | 32 | ICI | 0.055978729 | 0.029237719 | 0.114677927 |
| Refined PPS model | ethnicity | East_Asian | 229 | 138 | 91 | C | 0.825449912 | 0.766984788 | 0.874560902 |
| Refined PPS model | ethnicity | East_Asian | 229 | 138 | 91 | BalancedAccuracy | 0.764452938 | 0.706115195 | 0.817950466 |
| Refined PPS model | ethnicity | East_Asian | 229 | 138 | 91 | Sensitivity | 0.869565217 | 0.810201599 | 0.923076923 |
| Refined PPS model | ethnicity | East_Asian | 229 | 138 | 91 | Specificity | 0.659340659 | 0.56097561 | 0.752812293 |
| Refined PPS model | ethnicity | East_Asian | 229 | 138 | 91 | PPV | 0.794701987 | 0.727819601 | 0.856164384 |
| Refined PPS model | ethnicity | East_Asian | 229 | 138 | 91 | NPV | 0.769230769 | 0.674418605 | 0.855421687 |
| Refined PPS model | ethnicity | East_Asian | 229 | 138 | 91 | Intercept | -0.084523934 | -0.408034506 | 0.257146363 |
| Refined PPS model | ethnicity | East_Asian | 229 | 138 | 91 | Slope | 0.936851955 | 0.716664364 | 1.224546989 |
| Refined PPS model | ethnicity | East_Asian | 229 | 138 | 91 | Brier | 0.16025862 | 0.133850156 | 0.190106051 |
| Refined PPS model | ethnicity | East_Asian | 229 | 138 | 91 | ICI | 0.023443099 | 0.018324031 | 0.081398737 |
| Refined PPS model | ethnicity | Indigenous_Aboriginal | 22 | 19 | 3 | C | 0.754385965 | 0.523809524 | 0.952380952 |
| Refined PPS model | ethnicity | Indigenous_Aboriginal | 22 | 19 | 3 | BalancedAccuracy | 0.447368421 | 0.368421053 | 0.5 |
| Refined PPS model | ethnicity | Indigenous_Aboriginal | 22 | 19 | 3 | Sensitivity | 0.894736842 | 0.736842105 | 1 |
| Refined PPS model | ethnicity | Indigenous_Aboriginal | 22 | 19 | 3 | Specificity | 0 | 0 | 0 |
| Refined PPS model | ethnicity | Indigenous_Aboriginal | 22 | 19 | 3 | PPV | 0.85 | 0.666666667 | 0.952380952 |
| Refined PPS model | ethnicity | Indigenous_Aboriginal | 22 | 19 | 3 | NPV | 0 | 0 | 0 |
| Refined PPS model | ethnicity | Indigenous_Aboriginal | 22 | 19 | 3 | Intercept | 0.196281845 | -1.384212857 | 2.425852723 |
| Refined PPS model | ethnicity | Indigenous_Aboriginal | 22 | 19 | 3 | Slope | 0.359673919 | -0.112950367 | 2.776661741 |
| Refined PPS model | ethnicity | Indigenous_Aboriginal | 22 | 19 | 3 | Brier | 0.145397007 | 0.052651632 | 0.270601209 |
| Refined PPS model | ethnicity | Indigenous_Aboriginal | 22 | 19 | 3 | ICI | 0.10781208 | 0.065797423 | 0.329513414 |
| Refined PPS model | ethnicity | Mixed | 175 | 141 | 34 | C | 0.834376304 | 0.753377298 | 0.902765456 |
| Refined PPS model | ethnicity | Mixed | 175 | 141 | 34 | BalancedAccuracy | 0.688151856 | 0.603462662 | 0.770767024 |
| Refined PPS model | ethnicity | Mixed | 175 | 141 | 34 | Sensitivity | 0.964539007 | 0.932825736 | 0.992753623 |
| Refined PPS model | ethnicity | Mixed | 175 | 141 | 34 | Specificity | 0.411764706 | 0.25 | 0.571428571 |
| Refined PPS model | ethnicity | Mixed | 175 | 141 | 34 | PPV | 0.871794872 | 0.816993464 | 0.920252509 |
| Refined PPS model | ethnicity | Mixed | 175 | 141 | 34 | NPV | 0.736842105 | 0.529334365 | 0.928571429 |
| Refined PPS model | ethnicity | Mixed | 175 | 141 | 34 | Intercept | -0.01774251 | -0.475164732 | 0.482489145 |
| Refined PPS model | ethnicity | Mixed | 175 | 141 | 34 | Slope | 0.880981089 | 0.620304654 | 1.291146108 |
| Refined PPS model | ethnicity | Mixed | 175 | 141 | 34 | Brier | 0.113997545 | 0.083401014 | 0.146466085 |
| Refined PPS model | ethnicity | Mixed | 175 | 141 | 34 | ICI | 0.034769312 | 0.022113304 | 0.09492475 |
| Refined PPS model | ethnicity | Pacific_Islander | 5 | 5 | 0 | C | / | / | / |
| Refined PPS model | ethnicity | Pacific_Islander | 5 | 5 | 0 | BalancedAccuracy | / | / | / |
| Refined PPS model | ethnicity | Pacific_Islander | 5 | 5 | 0 | Sensitivity | / | / | / |
| Refined PPS model | ethnicity | Pacific_Islander | 5 | 5 | 0 | Specificity | / | / | / |
| Refined PPS model | ethnicity | Pacific_Islander | 5 | 5 | 0 | PPV | / | / | / |
| Refined PPS model | ethnicity | Pacific_Islander | 5 | 5 | 0 | NPV | / | / | / |
| Refined PPS model | ethnicity | Pacific_Islander | 5 | 5 | 0 | Intercept | / | / | / |
| Refined PPS model | ethnicity | Pacific_Islander | 5 | 5 | 0 | Slope | / | / | / |
| Refined PPS model | ethnicity | Pacific_Islander | 5 | 5 | 0 | Brier | / | / | / |
| Refined PPS model | ethnicity | Pacific_Islander | 5 | 5 | 0 | ICI | / | / | / |
| Refined PPS model | ethnicity | South_Asian | 127 | 78 | 49 | C | 0.808477237 | 0.723439799 | 0.885636892 |
| Refined PPS model | ethnicity | South_Asian | 127 | 78 | 49 | BalancedAccuracy | 0.748430141 | 0.672207377 | 0.823995996 |
| Refined PPS model | ethnicity | South_Asian | 127 | 78 | 49 | Sensitivity | 0.884615385 | 0.809510433 | 0.950015432 |
| Refined PPS model | ethnicity | South_Asian | 127 | 78 | 49 | Specificity | 0.612244898 | 0.473660287 | 0.744186047 |
| Refined PPS model | ethnicity | South_Asian | 127 | 78 | 49 | PPV | 0.784090909 | 0.696629213 | 0.869047619 |
| Refined PPS model | ethnicity | South_Asian | 127 | 78 | 49 | NPV | 0.769230769 | 0.628571429 | 0.892857143 |
| Refined PPS model | ethnicity | South_Asian | 127 | 78 | 49 | Intercept | -0.121693451 | -0.570491995 | 0.312753224 |
| Refined PPS model | ethnicity | South_Asian | 127 | 78 | 49 | Slope | 0.873445808 | 0.553793869 | 1.38105977 |
| Refined PPS model | ethnicity | South_Asian | 127 | 78 | 49 | Brier | 0.163552768 | 0.123706872 | 0.204176056 |
| Refined PPS model | ethnicity | South_Asian | 127 | 78 | 49 | ICI | 0.04922749 | 0.031923179 | 0.12398839 |
| Refined PPS model | ethnicity | Southeast_Asia | 119 | 92 | 27 | C | 0.855877617 | 0.765690419 | 0.927193235 |
| Refined PPS model | ethnicity | Southeast_Asia | 119 | 92 | 27 | BalancedAccuracy | 0.721215781 | 0.622891517 | 0.813193333 |
| Refined PPS model | ethnicity | Southeast_Asia | 119 | 92 | 27 | Sensitivity | 0.923913043 | 0.866666667 | 0.976750869 |
| Refined PPS model | ethnicity | Southeast_Asia | 119 | 92 | 27 | Specificity | 0.518518519 | 0.333333333 | 0.7 |
| Refined PPS model | ethnicity | Southeast_Asia | 119 | 92 | 27 | PPV | 0.867346939 | 0.79787234 | 0.930693069 |
| Refined PPS model | ethnicity | Southeast_Asia | 119 | 92 | 27 | NPV | 0.666666667 | 0.444444444 | 0.866666667 |
| Refined PPS model | ethnicity | Southeast_Asia | 119 | 92 | 27 | Intercept | 0.072286146 | -0.425064272 | 0.652025491 |
| Refined PPS model | ethnicity | Southeast_Asia | 119 | 92 | 27 | Slope | 1.002407316 | 0.674379694 | 1.59774653 |
| Refined PPS model | ethnicity | Southeast_Asia | 119 | 92 | 27 | Brier | 0.117430926 | 0.08411955 | 0.153405066 |
| Refined PPS model | ethnicity | Southeast_Asia | 119 | 92 | 27 | ICI | 0.034432754 | 0.02427104 | 0.100919557 |
| Refined PPS model | ethnicity | West_Central_Asia_or_Middle_Eastern | 60 | 52 | 8 | C | 0.882211538 | 0.75308642 | 0.987654321 |
| Refined PPS model | ethnicity | West_Central_Asia_or_Middle_Eastern | 60 | 52 | 8 | BalancedAccuracy | 0.774038462 | 0.591447829 | 0.962979798 |
| Refined PPS model | ethnicity | West_Central_Asia_or_Middle_Eastern | 60 | 52 | 8 | Sensitivity | 0.923076923 | 0.846078431 | 0.981481481 |
| Refined PPS model | ethnicity | West_Central_Asia_or_Middle_Eastern | 60 | 52 | 8 | Specificity | 0.625 | 0.25 | 1 |
| Refined PPS model | ethnicity | West_Central_Asia_or_Middle_Eastern | 60 | 52 | 8 | PPV | 0.941176471 | 0.872340426 | 1 |
| Refined PPS model | ethnicity | West_Central_Asia_or_Middle_Eastern | 60 | 52 | 8 | NPV | 0.555555556 | 0.222222222 | 0.888888889 |
| Refined PPS model | ethnicity | West_Central_Asia_or_Middle_Eastern | 60 | 52 | 8 | Intercept | 0.30337585 | -0.562139328 | 1.429076924 |
| Refined PPS model | ethnicity | West_Central_Asia_or_Middle_Eastern | 60 | 52 | 8 | Slope | 1.009824215 | 0.584848689 | 2.713314596 |
| Refined PPS model | ethnicity | West_Central_Asia_or_Middle_Eastern | 60 | 52 | 8 | Brier | 0.075604885 | 0.033690899 | 0.12621573 |
| Refined PPS model | ethnicity | West_Central_Asia_or_Middle_Eastern | 60 | 52 | 8 | ICI | 0.048562991 | 0.039217326 | 0.137271444 |
| Refined PPS model | ethnicity | White | 1192 | 924 | 268 | C | 0.89884183 | 0.879282432 | 0.918024031 |
| Refined PPS model | ethnicity | White | 1192 | 924 | 268 | BalancedAccuracy | 0.768147897 | 0.739319946 | 0.800249798 |
| Refined PPS model | ethnicity | White | 1192 | 924 | 268 | Sensitivity | 0.931818182 | 0.915491073 | 0.947651144 |
| Refined PPS model | ethnicity | White | 1192 | 924 | 268 | Specificity | 0.604477612 | 0.548631856 | 0.666666667 |
| Refined PPS model | ethnicity | White | 1192 | 924 | 268 | PPV | 0.890382627 | 0.872348006 | 0.909959462 |
| Refined PPS model | ethnicity | White | 1192 | 924 | 268 | NPV | 0.72 | 0.66186432 | 0.778735839 |
| Refined PPS model | ethnicity | White | 1192 | 924 | 268 | Intercept | -0.001496957 | -0.163827993 | 0.16682529 |
| Refined PPS model | ethnicity | White | 1192 | 924 | 268 | Slope | 1.140323065 | 1.024201231 | 1.298348951 |
| Refined PPS model | ethnicity | White | 1192 | 924 | 268 | Brier | 0.102010852 | 0.09162375 | 0.112395589 |
| Refined PPS model | ethnicity | White | 1192 | 924 | 268 | ICI | 0.014425382 | 0.01121947 | 0.032915971 |

**eTable 14**. Leave-one-site-out cross validation.

| **site** | **N subjects** | **N CHR-P** | **C_index** |
| --- | --- | --- | --- |
| BI | 33 | 28 | 0.929 |
| BM | 38 | 24 | 0.961 |
| CA | 48 | 33 | 0.964 |
| CG | 51 | 36 | 0.937 |
| CM | 39 | 30 | 0.956 |
| CP | 41 | 30 | 0.767 |
| GA | 49 | 34 | 0.888 |
| GW | 35 | 21 | 0.997 |
| HA | 29 | 27 | 0.889 |
| HK | 50 | 36 | 0.762 |
| IR | 50 | 38 | 0.818 |
| JE | 47 | 34 | 0.975 |
| KC | 37 | 23 | 0.904 |
| LA | 54 | 42 | 0.867 |
| LS | 27 | 18 | 0.957 |
| MA | 49 | 35 | 0.884 |
| ME | 677 | 577 | 0.836 |
| MT | 34 | 27 | 0.799 |
| MU | 17 | 11 | 1 |
| NC | 54 | 44 | 0.827 |
| NL | 36 | 28 | 0.848 |
| NN | 30 | 23 | 0.801 |
| OH | 6 | 4 | 1 |
| OR | 45 | 31 | 0.892 |
| PA | 59 | 40 | 0.763 |
| PI | 29 | 22 | 0.942 |
| PV | 44 | 34 | 0.976 |
| SD | 36 | 25 | 0.935 |
| SF | 53 | 38 | 0.9 |
| SG | 44 | 33 | 0.713 |
| SH | 19 | 11 | 0.773 |
| SI | 39 | 28 | 0.899 |
| SL | 16 | 9 | 1 |
| ST | 22 | 10 | 0.633 |
| TE | 51 | 34 | 0.849 |
| UR | 22 | 13 | 1 |
| WU | 54 | 40 | 0.866 |
| YA | 97 | 71 | 0.888 |
| overall | 2161 | 1642 | mean±sd=0.884±0.089 |

**eTable 15**. Performance metrics of the refined PPS model excluding some of the predictors (sensitivity analyses).

|  | **PPS refined model**  **(excluding parental psychosis and age)** | | **PPS refined model**  **(excluding gender and ethnicity)** | |
| --- | --- | --- | --- | --- |
|  | **Mean** | **CI** | **Mean** | **CI** |
| **C-Index** | 0.873 | (0.856-0.889) | 0.872 | (0.856-0.887) |
| **Balanced Accuracy** | 0.749 | (0.726-0.770) | 0.754 | (0.731-0.775) |
| **Sensitivity** | 0.931 | (0.919-0.943) | 0.926 | (0.913-0.938) |
| **Specificity** | 0.567 | (0.524-0.608) | 0.582 | (0.538-0.624) |
| **PPV** | 0.872 | (0.856-0.887) | 0.875 | (0.860-0.890) |
| **NPV** | 0.723 | (0.679-0.765) | 0.712 | (0.666-0.755) |
| **Calibration Intercept** | 0.005 | (-0.109-0.124) | -0.001 | (-0.118-0.117) |
| **Calibration Slope** | 1.046 | (0.952-1.142) | 1.008 | (0.917-0.117) |
| **Brier Score** | 0.115 | (0.107-0.124) | 0.115 | (0.107-0.124) |
| **ICI** | 0.016 | (0.008-0.027) | 0.021 | (0.011-0.032) |

**eFigure 2**. Calibration plots per each of the three PPS models.

^a^Calibration plot of the recalibrated PPS original model (predictor set 1), resulting from the external validation of the logistic regression coefficients derived from the EU-GEI study and tested on the AMP SCZ dataset.

^b^Calibration plot of the retrained PPS original model (predictor set 1), with no a priori coefficients.

^c^Calibration plot of the refined PPS model (predictor set 2).

**eTable 16**. Logistic regression coefficients of the refined PPS model*.

| **Predictor** | **Coefficient_raw** | **Coefficient_standardized** |
| --- | --- | --- |
| loneliness | 0.324877407 | 2.014728031 |
| father_psychosis | 1.600466336 | 1.600466336 |
| genderOther | 1.148181264 | 1.148181264 |
| mother_psychosis | 1.060101693 | 1.060101693 |
| father_other_mental_illness | 1.010843226 | 1.010843226 |
| emotional_abuse | 0.076489193 | 0.849240237 |
| mother_other_mental_illness | 0.838518609 | 0.838518609 |
| genderNon-binary | 0.826187286 | 0.826187286 |
| parent_ses | 0.630780345 | 0.630780345 |
| anhedonia | 0.022754415 | 0.577662885 |
| tobacco_daily | 0.506079923 | 0.506079923 |
| ethnicityWest_Central_Asia_or_Middle_Eastern | 0.435294218 | 0.435294218 |
| cannabis_daily | 0.343325579 | 0.343325579 |
| handedness | 0.296881164 | 0.296881164 |
| sexual_abuse | 0.03853732 | 0.284240821 |
| adult_life_events | 0.212897024 | 0.212897024 |
| physical_abuse | 0.018086155 | 0.120971637 |
| physical_neglect | 0.018637292 | 0.114930789 |
| emotional_neglect | 0.004186616 | 0.040270499 |
| genderFemale | -0.01298237 | -0.01298237 |
| premorbid_iq | -0.002974369 | -0.075377504 |
| second_gen_migrant | -0.082247148 | -0.082247148 |
| neighborhood_quality | -0.00926443 | -0.108245927 |
| first_gen_migrant | -0.272502019 | -0.272502019 |
| age | -0.038983334 | -0.299405741 |
| ethnicitySouth_Asian | -0.321018527 | -0.321018527 |
| ethnicityEast_Asian | -0.622587238 | -0.622587238 |
| (Intercept) | -3.753064876 | -3.753064876 |

^a^Raw coefficients = logistic regression coefficients derived from the 5-fold cross validation (CV) procedure using the final set of predictors.

^b^Standardized coefficients = same coefficients as raw values for binary predictors; values multiplied by two standard deviations for continuous predictors

*Categorical predictors were dummy-coded; coefficients refer to contrasts vs reference category.

**eTable 17.** Dropped or shrunk-to-zero variables within the refined PPS model.

| **Variable** | **Reason** |
| --- | --- |
| paternal age | >20% missing |
| childhood social withdrawal | >20% missing |
| genderMale | Shrunk to zero |
| ethnicityBlack | Shrunk to zero |
| ethnicityIndigenous Aboriginal | Shrunk to zero |
| ethnicityMixed | Shrunk to zero |
| ethnicityPacific Islander | Shrunk to zero |
| ethnicitySoutheast Asia | Shrunk to zero |
| North African first generation migrant | Shrunk to zero |
| North African second generation migrant | Shrunk to zero |

**eTable 18**. Logistic regression coefficients of the original PPS model (used for the external validation) and its retrained version within the AMP®SCZ dataset.

| **Predictor** | **Coefficient** | |
| --- | --- | --- |
|  | **PPS original model** | **PPS original model (retrained)** |
| (Intercept) | -0.6516721 | -1.002358895 |
| ethnicityOther | -0.172119 | -0.026669103 |
| first_gen_migrant | -0.09327723 | -0.382312399 |
| handedness | Shrunk_to_zero | 0.064553877 |
| parent_smi | 0.7848041 | 1.29827224 |
| adult_life_events | 0.4013517 | 0.90258256 |
| tobacco_daily | 0.4323031 | 0.363205288 |
| heavy_cannabis_use | 0.6674957 | 0.305175495 |
| parent_ses | 0.6252665 | 0.751967824 |
| childhood_trauma | 1.485927 | 1.579965467 |
| anhedonia | 1.99204 | 1.046959772 |
| ethnicityWhite | 0.3725069 | Shrunk_to_zero |
| ethnicityBlack | Shrunk_to_zero | Shrunk_to_zero |
| second_gen_migrant | 0.07709335 | Shrunk_to_zero |
| north_african_first_gen_migrant | 5.65E-16 | Shrunk_to_zero |
| north_african_second_gen_migrant | 1.025347 | Shrunk_to_zero |
| male_25_35_years | 0.06657729 | Shrunk_to_zero |

**eFigure 3.** Association of PPS scores^a^ with subthreshold positive symptoms and socio-occupational functioning restricted to CHR individuals*.

^a^PPS scores = each participant’s PPS score based on the refined PPS model. More specifically, PPS scores were obtained by the sum of each predictor (i.e., predictor set 2) multiplied by its associated logistic regression coefficient; ρ = Spearman rank-based correlation coefficient measuring the strength and direction of a monotonic relationship between two variables.

Positive symptom severity was measured by the PSYCHS; socio-occupational functioning was measured by the SOFAS.

*both Spearman correlation coefficients were statistically significant at the p<.001 level.

**eFigure 4.** Decision curve analysis (DCA) comparing the three PPS models within the clinical population.

**eFigure 5.** Decision curve analysis (DCA) comparing the three PPS models within the general population.

**eDiscussion**

Gender minority status also emerged as a positive predictor of CHR status. This may suggest an increased accumulation of environmental risk factors such as minority stress, social marginalization and discrimination, and elevated exposure to trauma^17, 18^. This finding highlights the importance of addressing stigma and discrimination faced by these individuals by giving voice to their subjective experiences, as recently done in a co-written article led by some of these authors, which has summarized the lived experiences of trans-identifying people^19^.

Conversely, South and East Asian ethnicity showed negative coefficients in the refined classification model. These ethnic groups were distributed across multiple sites, and Asian sites did not show atypical CHR/CC ratios, suggesting that this effect is unlikely to be solely driven by site composition. By contrast, it may reflect reduced exposure to known environmental risks and/or the presence of resilience-promoting sociocultural factors. For instance, South and East Asian populations tend to exhibit extremely low rates of adolescent cannabis use due to cultural norms and strict drug enforcement^20^. In addition, Asian communities outside Asia often feature high ethnic density and cohesive family structures^21^, which may buffer against discrimination stress^22^ and enhance social support^23^, thereby reducing psychosis risk^24-26^. However, these interpretations remain speculative in the present cross-sectional classification analysis and require further investigation in external datasets.

At the methodological level, there is converging evidence that predictor variables that may be associated with biases (e.g., ethnicity) should be included in prediction models, and that excluding them from models can inflate potential stigma and discrimination, leaving them unaddressed^27, 28^. Accordingly, we have conducted sensitivity analyses by removing these predictors, and the model retained good performance (eTable 12), thus demonstrating a negligible impact of such predictors.

**eReferences.**

1.	Gershon ES, Maxwell ME. Family Interview for Genetic Studies (FIGS). Bethesda, MD, USA; 1992.

2.	Brugha TS, Cragg D. The List of Threatening Experiences: the reliability and validity of a brief life events questionnaire. Acta Psychiatr Scand. 1990;82(1):77-81.10.1111/j.1600-0447.1990.tb01360.x

3.	Group WAW. The Alcohol, Smoking and Substance Involvement Screening Test (ASSIST): development, reliability and feasibility. Addiction. 2002;97(9):1183-94.10.1046/j.1360-0443.2002.00185.x

4.	Hagborg JM, Kalin T, Gerdner A. The Childhood Trauma Questionnaire-Short Form (CTQ-SF) used with adolescents - methodological report from clinical and community samples. J Child Adolesc Trauma. 2022;15(4):1199-213.10.1007/s40653-022-00443-8

5.	De Jong Gierveld J, Van Tilburg TG. A 6-Item Scale for Overall, Emotional, and Social Loneliness: Confirmatory Tests on Survey Data. Research on Aging. 2006;28(5):582–98.10.1177/0164027506289723

6.	Kirkbride JB, Boydell J, Ploubidis GB, Morgan C, Dazzan P, McKenzie K, et al. Testing the association between the incidence of schizophrenia and social capital in an urban area. Psychol Med. 2008;38(8):1083-94.10.1017/s0033291707002085

7.	Chan RC, Shi YF, Lai MK, Wang YN, Wang Y, Kring AM. The Temporal Experience of Pleasure Scale (TEPS): exploration and confirmation of factor structure in a healthy Chinese sample. PLoS One. 2012;7(4):e35352.10.1371/journal.pone.0035352

8.	Wilkinson GS, Robertson GJ. Wide Range Achievement Test–Fifth Edition (WRAT-5). Lutz, FL; 2017.

9.	Cannon-Spoor HE, Potkin SG, Wyatt RJ. Measurement of premorbid adjustment in chronic schizophrenia. Schizophr Bull. 1982;8(3):470-84.10.1093/schbul/8.3.470

10.	Riley RD, Ensor J, Snell KIE, Harrell FE, Jr., Martin GP, Reitsma JB, et al. Calculating the sample size required for developing a clinical prediction model. Bmj. 2020;368:m441.10.1136/bmj.m441

11.	Oliver D, Arribas M, Logeswaran Y, Fusar-Poli L, Kempton MJ, Hedges EP, et al. Psychosis polyrisk score (PPS) and polygenic risk score to improve detection and prognosis in individuals at clinical high risk for psychosis. Schizophr Bull. 2026;In press

12.	Yung AR, Yuen HP, McGorry PD, Phillips LJ, Kelly D, Dell'Olio M, et al. Mapping the onset of psychosis: the Comprehensive Assessment of At-Risk Mental States. Aust N Z J Psychiatry. 2005;39(11-12):964-71.10.1080/j.1440-1614.2005.01714.x

13.	Miller TJ, Cicchetti D, Markovich PJ, McGlashan TH, Woods SW. The SIPS Screen: a brief self-report screen to detect the schizophrenia prodrome. Schizophrenia Research. 2004;70:78

14.	Woods SW, Parker S, Kerr MJ, Walsh BC, Wijtenburg SA, Prunier N, et al. Development of the PSYCHS: Positive SYmptoms and Diagnostic Criteria for the CAARMS Harmonized with the SIPS. medRxiv. 2023.10.1101/2023.04.29.23289226

15.	Shabani A, Masoumian S, Zamirinejad S, Hejri M, Pirmorad T, Yaghmaeezadeh H. Psychometric properties of Structured Clinical Interview for DSM-5 Disorders-Clinician Version (SCID-5-CV). Brain Behav. 2021;11(5):e01894.10.1002/brb3.1894

16.	Morosini PL, Magliano L, Brambilla L, Ugolini S, Pioli R. Development, reliability and acceptability of a new version of the DSM-IV Social and Occupational Functioning Assessment Scale (SOFAS) to assess routine social functioning. Acta Psychiatr Scand. 2000;101(4):323-9

17.	Barr SM, Roberts D, Thakkar KN. Psychosis in transgender and gender non-conforming individuals: A review of the literature and a call for more research. Psychiatry Res. 2021;306:114272.10.1016/j.psychres.2021.114272

18.	Termorshuizen F, de Vries ALC, Wiepjes CM, Selten JP. The risk of psychosis for transgender individuals: a Dutch national cohort study. Psychol Med. 2023;53(16):7923-32.10.1017/s0033291723002088

19.	Paolo Fusar-Poli, Andrés Estradé, René Rosfort, Annemijn van der Schaar, Jordan, Searle-Barnes, et al. The lived experience of trans persons: a bottom-up review co-written by experts by

experience and academics World Psychiatry. 2026;In press

20.	Lee TY, Kwon JS. Psychosis research in Asia: advantage from low prevalence of cannabis use. NPJ Schizophr. 2016;2:1.10.1038/s41537-016-0002-4

21.	Zhang Q, Metcalf SS, Palmer HD, Northridge ME. Spatial Analysis of Chinese American Ethnic Enclaves and Community Health Indicators in New York City. Front Public Health. 2022;10:815169.10.3389/fpubh.2022.815169

22.	Juang LP, Alvarez AA. Discrimination and adjustment among Chinese American adolescents: family conflict and family cohesion as vulnerability and protective factors. Am J Public Health. 2010;100(12):2403-9.10.2105/ajph.2009.185959

23.	Tseng M, Walton E, Handorf E, Fang CY. Ethnic density, social support, and loneliness among Chinese immigrants in Philadelphia. Wellbeing Space Soc. 2021;2.10.1016/j.wss.2021.100050

24.	Anglin DM, Espinosa A, Addington J, Cadenhead KS, Cannon TD, Cornblatt BA, et al. Association of Childhood Area-Level Ethnic Density and Psychosis Risk Among Ethnoracial Minoritized Individuals in the US. JAMA Psychiatry. 2023;80(12):1226-34.10.1001/jamapsychiatry.2023.2841

25.	Baker SJ, Jackson M, Jongsma H, Saville CWN. The ethnic density effect in psychosis: a systematic review and multilevel meta-analysis. Br J Psychiatry. 2021;219(6):632-43.10.1192/bjp.2021.96

26.	Veling W, Susser E, van Os J, Mackenbach JP, Selten JP, Hoek HW. Ethnic density of neighborhoods and incidence of psychotic disorders among immigrants. Am J Psychiatry. 2008;165(1):66-73.10.1176/appi.ajp.2007.07030423

27.	Diao JA, Shi I, Murthy VL, Buckley TA, Patel CJ, Pierson E, et al. Projected Changes in Statin and Antihypertensive Therapy Eligibility With the AHA PREVENT Cardiovascular Risk Equations. Jama. 2024;332(12):989-1000.10.1001/jama.2024.12537

28.	Khor S, Haupt EC, Hahn EE, Lyons LJL, Shankaran V, Bansal A. Racial and Ethnic Bias in Risk Prediction Models for Colorectal Cancer Recurrence When Race and Ethnicity Are Omitted as Predictors. JAMA Netw Open. 2023;6(6):e2318495.10.1001/jamanetworkopen.2023.18495
